# The neural signature of reality‐monitoring: A meta‐analysis of functional neuroimaging studies

**DOI:** 10.1002/hbm.26387

**Published:** 2023-05-29

**Authors:** Layla Lavallé, Jérôme Brunelin, Renaud Jardri, Frédéric Haesebaert, Marine Mondino

**Affiliations:** ^1^ Université Claude Bernard Lyon 1, CNRS, INSERM, Centre de Recherche en Neurosciences de Lyon CRNL U1028 UMR5292, PSYR2 Bron France; ^2^ CH le Vinatier Bron France; ^3^ Université de Lille, INSERM U‐1172, Lille Neurosciences & Cognition, Plasticity & Subjectivity Team Lille France

**Keywords:** coordinate‐based meta‐analysis, fMRI, reality‐monitoring, self‐monitoring

## Abstract

Distinguishing imagination and thoughts from information we perceived from the environment, a process called reality‐monitoring, is important in everyday situations. Although reality monitoring seems to overlap with the concept of self‐monitoring, which allows one to distinguish self‐generated actions or thoughts from those generated by others, the two concepts remain largely separate cognitive domains and their common brain substrates have received little attention. We investigated the brain regions involved in these two cognitive processes and explored the common brain regions they share. To do this, we conducted two separate coordinate‐based meta‐analyses of functional magnetic resonance imaging studies assessing the brain regions involved in reality‐ and self‐monitoring. Few brain regions survived threshold‐free cluster enhancement family‐wise multiple comparison correction (*p* < .05), likely owing to the small number of studies identified. Using uncorrected statistical thresholds recommended by Signed Differential Mapping with Permutation of Subject Images, the meta‐analysis of reality‐monitoring studies (*k* = 9 studies including 172 healthy subjects) revealed clusters in the lobule VI of the cerebellum, the right anterior medial prefrontal cortex and anterior thalamic projections. The meta‐analysis of self‐monitoring studies (*k* = 12 studies including 192 healthy subjects) highlighted the involvement of a set of brain regions including the lobule VI of the left cerebellum and fronto‐temporo‐parietal regions. We showed with a conjunction analysis that the lobule VI of the cerebellum was consistently engaged in both reality‐ and self‐monitoring. The current findings offer new insights into the common brain regions underlying reality‐monitoring and self‐monitoring, and suggest that the neural signature of the self that may occur during self‐production should persist in memories.

## Graphical abstract

We used a coordinate‐based meta‐analytic approach to investigate the neural substrates of reality‐monitoring. We reported the involvement of lobule VI of the cerebellum, the right anterior medial prefrontal cortex and anterior thalamic projections. We then conducted a conjunction analysis to detect brain regions common to reality‐monitoring and self‐monitoring and identified that the lobule VI of the cerebellum is consistently engaged in both processes.

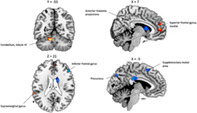

## 1 INTRODUCTION

How can we determine that memories come from real perceptions and not from imagination? The process of making attributions about the source of memories between internal sources (such as imagination) and external sources (such as perception) is called reality monitoring. Strong reality‐monitoring capacities are necessary in everyday life, for instance, to distinguish our mental imagery, or the events that we daydream about, from the events that actually occurred.

Reality monitoring has been theorized into the “source‐monitoring framework” by Johnson et al. (1993), who suggested that memories did not come with a label indicating their source; but that the source is rather determined based on several cues associated with the event such as the amount of perceptual details, contextual information and cognitive operations. According to the source‐monitoring framework, veridical perceptions include more and stronger sensory details, whereas imagination is under more top‐down cognitive control signals due to the rich cognitive operations involved in generating the mental experience. A higher‐order reality‐monitoring mechanism is then supposed to integrate information about sensory signals and cognitive control to make source attributions (Garrison et al., 2017; Johnson, 1997; Johnson et al., 1993).

Reality monitoring seems intrinsically tied to self‐monitoring, that is, the ability to distinguish self‐generated actions or thoughts from actions or thoughts generated by others, and more broadly to the concept of self‐agency, that is, the experience of being the agent of one's action or thought and the feeling that self‐productions are intentional and associated with a cognitive experience of voluntary control (Haggard, 2017). However, only a few studies investigated the relationship between the two processes. In one of them, Subramaniam et al. showed that the reality‐monitoring imagination/perception decision was correlated with self‐monitoring measures, which provides support for a unitary experience of self‐agency resulting from the ability to reliably predict the outcome of self‐generated actions (Subramaniam et al., 2018). Where and to what extend reality monitoring and self‐monitoring processes overlap in the brain remains unknown.

Several neuroimaging studies have tried to identify neural substrates of reality monitoring. A qualitative review of imaging studies highlighted the crucial role of the medial prefrontal cortex (mPFC) and especially its anterior part (amPFC) in distinguishing between the imagined or perceived origin of a signal (Simons et al., 2017). Activity in this area, located in the anterior portion of the mPFC, was observed regardless of stimulus type (i.e., voice, faces, objects). Any interpretation of the amPFC as the key structure for reality‐monitoring needs to be cautious because most of the included studies reported results that were bound to the scope of an a priori defined region of interest (ROI) in the amPFC. However, a causal role for the mPFC has also been established by using noninvasive brain stimulation to target the mPFC and improve reality‐monitoring performance (Subramaniam et al., 2020).

Regarding self‐monitoring, two previous meta‐analyses have revealed converging activations in the left inferior parietal lobule (IPL) and the right temporoparietal junction (TPJ), including the supramarginal gyrus, the angular gyrus, and the superior temporal gyrus, when confronted with externally derived information (Seghezzi et al., 2019; Sperduti et al., 2011). Their results when facing self‐generated information were more heterogeneous: Seghezzi et al. revealed activations in the left supplementary motor area, left posterior insula, right calcarine scissure, and right cerebellum while Sperduti et al. revealed activations in the bilateral postcentral gyrus, left precentral gyrus, and insula. These conflicting findings can be due to common methodological issues, such as the inclusion of small‐volume corrected (SVC) analyses that violate the assumption that all included experiments should be based on the same search and whole‐brain coverage. Moreover, their use of the *activation likelihood estimation* approach led them to analyze activations and deactivations in two separate analyses, which may lead to not counteract positive and negative differences in the same brain areas, potentially leading to voxels being detected as increased and decreased at the same time (Radua & Mataix‐Cols, 2012).

The current study aimed to determine whether reality‐monitoring and self‐monitoring recruit overlapping brain regions, thereby allowing us to deepen our understanding of their common underlying cognitive processes. Specifically, this study had three aims:

1. For the first time, we meta‐analyzed the neural substrates of reality‐monitoring. We expected that distinguishing imagination‐ from perception‐derived information would activate the amPFC.
2. We also updated the current knowledge regarding the substrates of self‐monitoring using a relatively unbiased meta‐analytic approach and strict inclusion criteria. Based on the two previous coordinate‐based meta‐analyses, we expected modulation of activity in the bilateral temporo‐parietal regions.
3. Third, we aimed to determine whether reality‐monitoring and self‐monitoring are associated with similar activations. Based on previous behavioral studies showing a strong correlation between reality‐monitoring and self‐monitoring behavioral scores, we argue that observing such spatial overlap would support the hypothesis of partially shared cognitive mechanisms between these two processes.

## 2 METHODS

This systematic review was performed according to the recommendations from the Preferred Reporting Items for Systematic Reviews and Meta‐Analyses (PRISMA) guidelines (Moher et al., 2009). The protocol was registered in PROSPERO (Chien et al., 2012) (registration number: CRD42020204113 on September 29, 2020).

### 2.1 Search strategy

The articles included in the meta‐analyses were retrieved using a systematic search strategy. We searched for articles published up until May 2022 without any starting date in the PubMed and ScienceDirect databases. We used the following terms for the reality‐monitoring meta‐analysis: (“source‐monitoring” OR “reality‐monitoring” OR “self‐related”) AND (“fMRI” OR “functional magnetic resonance imaging” OR “PET” OR “positron emission tomography” OR “neuroimaging”) and the following search terms for the self‐monitoring meta‐analysis: (“self‐monitoring” OR “agency” OR “self‐related”) AND (“fMRI” OR “PET” OR “neuroimaging”). We identified a total of 253 overlapping papers between these two searches. Additional relevant articles were retrieved by up and down ancestry search across all the selected articles. The “similar articles” function of PubMed was also employed, although no additional references were identified in this manner. Finally, we manually searched through review articles on reality‐monitoring, agency, self‐judgment, and self‐referential thinking to find additional topics falling into our inclusion criteria (Denny et al., 2012; Morin & Hamper, 2012; Seghezzi et al., 2019; Simons et al., 2017; Sperduti et al., 2011; van der Meer et al., 2010; van Veluw & Chance, 2014).

**FIGURE 1:**
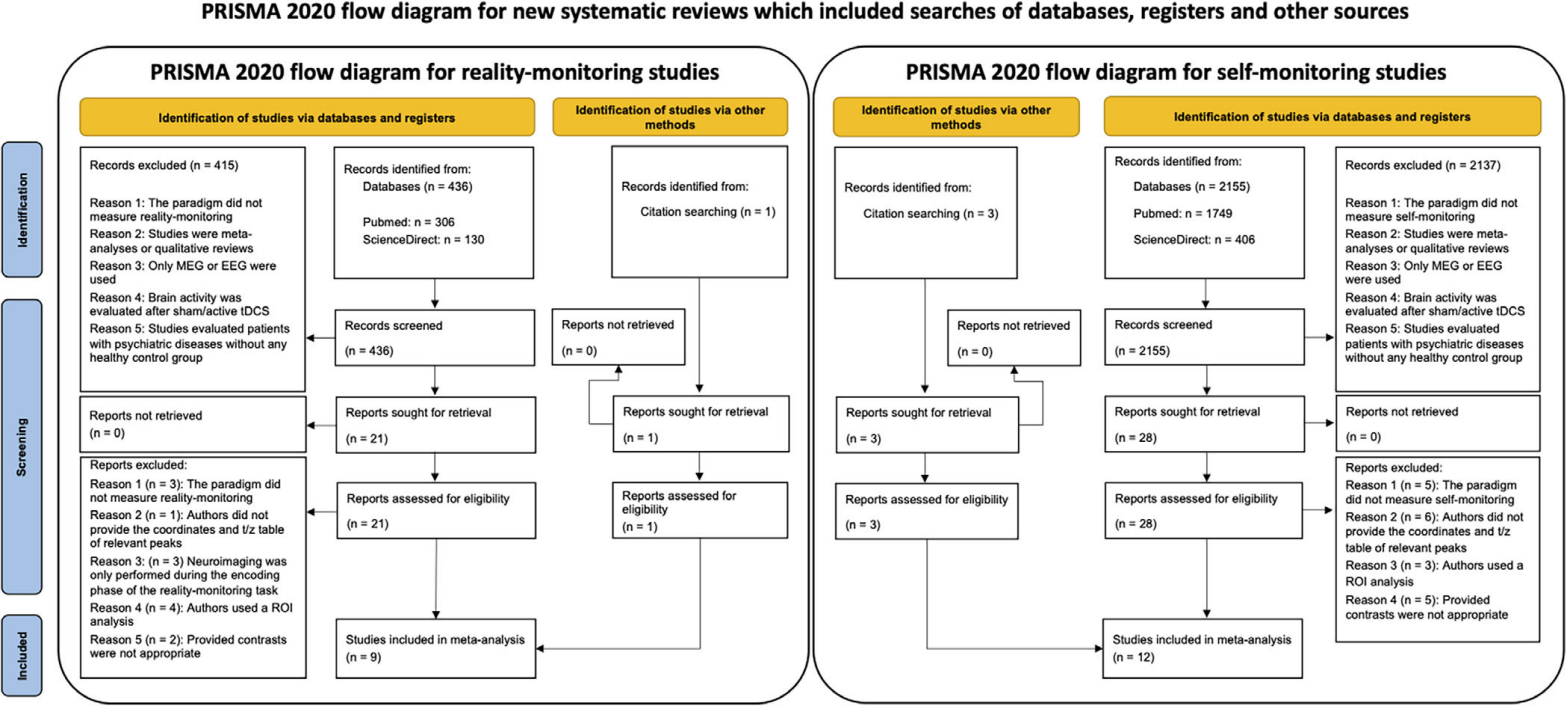
Preferred Reporting Items for Systematic Reviews and Meta‐Analyses (PRISMA) flow diagram of the literature search for the reality‐monitoring (left) and self‐monitoring (right) meta‐analyses.

The detailed process of article selection and the reasons for exclusions are depicted in the PRISMA flowcharts presented in Figure 1.

### 2.2 Eligibility

**FIGURE 2:**
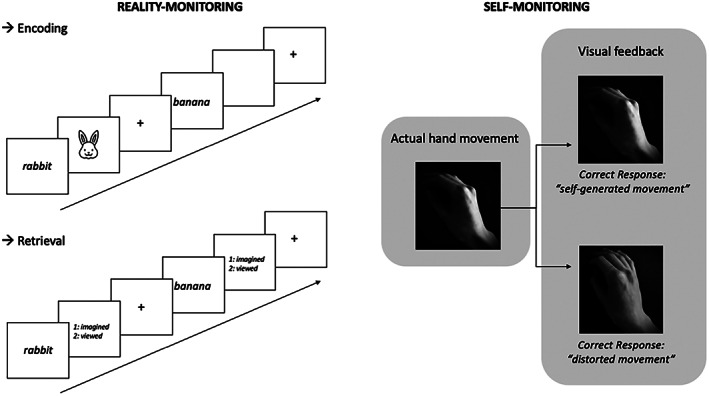
Examples of typical reality‐monitoring and self‐monitoring paradigms. Reality‐monitoring paradigms (left panel) typically consist of an encoding phase, in which participants are confronted with either perceived items or items that must be imagined (in the case of a visual reality‐monitoring paradigm, participants see a word and either see a corresponding picture or have to imagine a corresponding mental image). The encoding phase is followed by a retrieval phase, in which participants had to recall whether each item was actually perceived (seen) or only imagined. In typical self‐monitoring paradigms (right panel), participants perform a movement while receiving either congruent (e.g., visual feedback of their own movement) or incongruent feedback (e.g., visual feedback of a distorted movement). Participants are either asked to judge whether the movement was their own or not, or, in the case of implicit paradigms, no response is requested.

The inclusion criteria were as follows: (i) original articles were written in the English language and published in peer‐reviewed journals, (ii) healthy volunteers without any established clinical diagnosis of neurological or psychiatric disease were included, (iii) task‐related fMRI or PET contrasts were reported, and (iv) studies provided clear information regarding the task and used either the reality‐monitoring paradigm (i.e., a paradigm eliciting subjects to judge whether information was previously self‐generated or derived from outside) or a self‐monitoring paradigm (i.e., a paradigm eliciting subjects to make comparisons between sensory predictions and continuous sensory feedback). Typical reality‐monitoring and self‐monitoring paradigms are illustrated in Figure 2. (v) Concerning the reality‐monitoring meta‐analysis, neuroimaging explored brain activity during the retrieval phase of the task, (vi) studies conducted direct statistical comparisons between self‐ and nonself‐conditions (self > nonself; self < nonself), (vii) studies reported results from whole‐brain analyses with full‐brain coverage), (viii) studies reported x/y/z coordinates in either standard space, Talairach space or Montreal Neurological Institute (MNI) spaces, and (ix) studies reported Z‐statistics, t‐statistics or uncorrected *p*‐values.

The exclusion criteria were as follows: (i) studies explored brain activity only during the encoding phase of the reality‐monitoring paradigm, (ii) studies used SVC analyses, (iii) studies only reported ROI analysis, (iv) studies used data from subjects already included in other studies, (v) studies included fewer than five healthy subjects, and (vi) studies reporting other statistical comparisons (e.g., misattribution of the source vs. correct attribution).

Decisions on inclusion and data extraction were made independently by two authors (LL and MM).

### 2.3 Data extraction

For each selected article, the following demographic and task‐related information was extracted: sample size, gender, and age of subjects, imaging modality (fMRI or PET), detailed description of the design and the task used, and pertinent contrasts (*see* Table 1, Supplementary Table 1). Regarding gender information, it may be mentioned that several studies did not clearly report the number of male and female after exclusion of participants. For each dataset, we extracted x/y/z coordinates and Z‐ or t‐statistics or uncorrected *p*‐values (see Supplementary Table 2). Aggregated data were shared on the Open Science Framework (https://osf.io/7xm9t/?view_only=c92372b173c742f98fd2d54b3acee328). We also reported several MRI‐related acquisition and analysis parameters: the fMRI design (event‐related or block), the magnetic field strength, the number of acquired slices, slice thickness and gap, the field of view, the matrix size, the software used for analysis (SPM, FSL or other) and its version, the reference space (MNI or TAL), smoothing kernel, and the statistical threshold used (see Supplementary Table 3).

**TABLE 1:** Characteristics of the studies included in the reality‐monitoring (upper panel) and self‐monitoring (lower panel) meta‐analyses.

Author, year	*n*	Sex (% M)	Age (range)	Neuroimaging	Analysis software	Paradigm	Stimuli	Contrast of interest	Quality
Takahashi et al. (2002)	13	78.6^a^	(19–30)	fMRI	SPM99	Reality‐monitoring	Verbal	Self > nonself; self < nonself	0.69
Turner et al. (2008)	16	31.2^a^	26.2 (19–36)	fMRI	SPM2	Reality‐monitoring	Verbal	Self > nonself	0.78
King and Miller (2014)	20	45	28.1 (20–51)	fMRI	SPM5	Reality‐monitoring	Verbal	Self > nonself; self < nonself	0.53
Subramaniam et al. (2012)	15	68.7^a^	45	fMRI	SPM2	Reality‐monitoring	Verbal	Self > nonself	0.84
Lundstrom et al. (2003)	21	52.3	24 (20–28)	fMRI	SPM99	Reality‐monitoring	Verbal	Self > nonself	0.56
King and Miller (2017)	28	48.5^a^	21 (19–32)	fMRI	SPM8	Reality‐monitoring	Verbal + image	Self < nonself	0.91
King et al. (2015)	27	62.8^a^	26.4 (20–34)	fMRI	SPM8	Reality‐monitoring	Verbal + image	Self < nonself	0.91
Vinogradov et al. (2008)	8	50	28 (25–33)	fMRI	SPM2	Reality‐monitoring	Verbal	Self > nonself	0.53
Stephan‐Otto, Siddi, Senior, Muñoz‐Samons, et al. (2017)	24	38.7^a^	37.3	fMRI	SPM8	Reality‐monitoring	Verbal + image	Self < nonself	0.71
Tsakiris et al. (2010)	19	60^a^	24.8 (18–36)	fMRI	SPM5	Self‐monitoring	Action	Self > nonself; self < nonself	0.80
Uhlmann et al. (2020)	23	47.8	26.4 (20–35)	fMRI	SPM12	Self‐monitoring	Action	Self > nonself; self < nonself	0.56
Renes et al. (2015)	23	52.1	21.7	fMRI	SPM5	Self‐monitoring	Action	Self > nonself	0.87
Farrer and Frith (2002)	12	66.7	29	fMRI	SPM99	Self‐monitoring	Action	Self > nonself; self < nonself	0.67
Kontaris et al. (2009)	11	18.2	24	fMRI	BVQX 1.9	Self‐monitoring	Action	Self > nonself; self < nonself	0.60
Sasaki et al. (2018)	24	54.2	24.8	fMRI	SPM8	Self‐monitoring	Action	Self > nonself	0.77
Schnell et al. (2007)	15	100	29.49	fMRI	SPM2	Self‐monitoring	Action	Self < nonself	0.67
Jardri et al. (2007)	12	50	(25–29)	fMRI	BVQX 1.7.9	Self‐monitoring	Verbal	Self < nonself	0.40
Jardri, Pins, et al. (2011)	15	66.7	30.1	fMRI	BVQX 1.9	Self‐monitoring	Verbal	Self < nonself	0.80
Balslev et al. (2006)	15	46.7	(20–28)	fMRI	SPM2	Self‐monitoring	Action	Self < nonself	0.86
Farrer et al. (2008)	15	73.3	20.7	fMRI	SPM99	Self‐monitoring	Action	Self < nonself	0.60
Farrer et al. (2003)	8	100	34	PET	SPM99	Self‐monitoring	Action	Self > nonself; self < nonself	0.72

*Note*: Range of quality score: 0–1.

Abbreviations: BVQX, BrainVoyager QX; fMRI, functional magnetic resonance imaging; M/F, male/female; PET, positron emission tomography.

^a^ Studies mentioning the number of male and female before removal of participants from the fMRI analyses.

### 2.4 Quality assessment

A quality assessment score was also calculated based on the criteria used in the study by Tian et al. (2020) and the guidelines for reporting an fMRI study by Poldrack et al. (2008). The final checklist included 18 items evaluating, among other things, the subject sample, design specification, data acquisition, data preprocessing, statistical analyses, and reporting of conclusions (see Supplementary Table 4).

### 2.5 Seed‐based d mapping

Data were analyzed using seed‐based d mapping software (formerly *Signed Differential Mapping*) with Permutation of Subject Images (SDM‐PSI, version 6.21, https://www.sdmproject.com/). This voxel‐based method allowed us to summarize peak coordinates and statistical t‐maps from the multiple included studies to produce a whole‐brain summary of brain activity associated with self‐agency and had been extensively validated by previous meta‐analyses (Albajes‐Eizagirre, Solanes, & Radua, 2019; Albajes‐Eizagirre, Solanes, Vieta, & Radua, 2019; Radua & Mataix‐Cols, 2009; Radua, Mataix‐Cols, et al., 2012; Radua, Rubia, et al., 2014).

SDM‐PSI imputes the brain maps of statistical effects for each included study to conduct a standard random‐effect meta‐analysis that tests whether the effects are different from zero. This method was mainly chosen in the current study because it offers the key advantages of accounting for effect sizes and analyzing both positive and negative peaks in the same map, to counteract the effects of studies reporting opposite activation findings in the same areas (Radua & Mataix‐Cols, 2012). These properties have previously been shown to enhance the balance between the false and positive rate and increase reliability, particularly with a small number of included studies in the meta‐analysis (Bossier et al., 2018).

The procedure includes four main steps: data preparation, preprocessing, mean analysis, and complementary analyses (heterogeneity and publication bias analyses).

1. During data preparation, Z‐values were first converted into t‐values with the SDM statistics converter (https://www.sdmproject.com/utilities/?show=Statistics). Then, coordinates and t‐values were written in separate text files to be extracted by SDM. The t‐values obtained from the analysis of the “self < nonself” contrast were added with a negative sign corresponding to deactivation.
2. Then, data were preprocessed to convert t‐values for each peak of activation into Hedges' *g* effect size and their associated variance, thereby obtaining the maximum likely maps of the lower and upper bounds of potential effect sizes for each study.
3. During the main analysis, SDM allowed us to calculate the mean of the voxel values in the different studies. Hedge's g‐corrected effect sizes were calculated at the group level, and a random model was run with each study weighted by its variance and between‐study heterogeneity. Finally, the family‐wise error (FWE) rate was applied to correct for multiple comparisons. The default setting of 1000 permutations has been kept. The distribution of the maximum statistics obtained was then used to threshold the meta‐analysis images, resulting in a corrected *p*‐value map.
4. Finally, heterogeneity was studied by analyzing a map of *I* ^2^ statistics and potential publication bias was evaluated using funnel plots and Egger's tests. *I* ^2^ values are typically categorized as low, moderate, and high for values of 25, 50, and 75%, respectively.

We reported results using an uncorrected *p* < .005 threshold with a cluster extent = 20 voxels (Lieberman & Cunningham, 2009; Radua, Borgwardt, et al., 2012; Radua, Mataix‐Cols, et al., 2012). We also reported using FWE‐corrected *p* < .05 using the threshold‐free cluster enhancement (TFCE) approach (Dugré et al., 2020; Smith & Nichols, 2009). The use of two statistical thresholds is common in SDM meta‐analyses. A simulation comparing the results of meta‐analyses and mega‐analyses on the same data showed that the “liberal” uncorrected threshold of *p* < .005 optimally balance false‐positive and false‐negative rates (Radua, Mataix‐Cols, et al., 2012) and is thus recommended (Müller et al., 2018). An extent threshold of 20 voxels was applied, which is stricter than the threshold suggested by Radua et al. to exclude smaller clusters. TFCE FWE‐corrected results were reported to increase the specificity‐to‐sensitivity ratio. The corrected results as well as the reported effect‐sizes should be taken in consideration to judge the strength of evidence of a true effect (Müller et al., 2018). All activations/deactivations were reported in the MNI space. The regions listed in the results tables were labeled using the SDM stereotactic space (Radua, Grau, et al., 2014; Thiebaut de Schotten, Dell'Acqua, et al., 2011; Thiebaut de Schotten, Ffytche, et al., 2011) and the SPM12 Anatomy Toolbox v3.0 (Eickhoff et al., 2007).

Based on our aims and predictions, we carried out two sets of analyses:

1. We performed separate meta‐analyses to examine the neural substrate of both reality‐monitoring and self‐monitoring paradigms.
2. We conducted a conjunction analysis between the meta‐analytical map of reality‐monitoring and that of self‐monitoring using the multimodal analysis function (Radua et al., 2013) of SDM‐PSI to identify regions that were associated with both reality‐monitoring and self‐monitoring.

### 2.6 Reliability analyses

To test the robustness of the results, a jackknife sensitivity procedure was conducted (Radua & Mataix‐Cols, 2009). This analysis was carried out by successively repeating the mean analysis as many times as studies were included but discarding one different individual study at a time. Findings were considered highly replicable when significant brain regions remained significant in all the included studies.

### 2.7 Supplemental analysis: Comparison between paradigms

We conducted a supplemental analysis that directly compares changes in activity between reality‐monitoring and self‐monitoring studies. Results were reported using an uncorrected *p* < .005 threshold with a cluster extent = 20 voxels.

### 2.8 Supplemental analysis: Controlling the potential confounding

The potential influence of age and study quality on estimated activations/deactivations was further explored by meta‐regression using a linear random‐effect model. Results were considered statistically significant at FWE‐corrected a conservative threshold of *p* = .005 to reduce the risk of type 1 error related to multiple testing and minimize the detection of spurious relationships. Only brain regions also found in the main meta‐analyses were considered.

## 3 RESULTS

After the selection process and removal of duplicates, 9 studies met the criteria for inclusion in the reality‐monitoring meta‐analysis, including a total of 172 subjects. Then, 12 studies met the criteria for inclusion in the self‐monitoring meta‐analysis, including a total of 192 subjects.

### 3.1 Brain responses associated with reality‐monitoring

**FIGURE 3:**
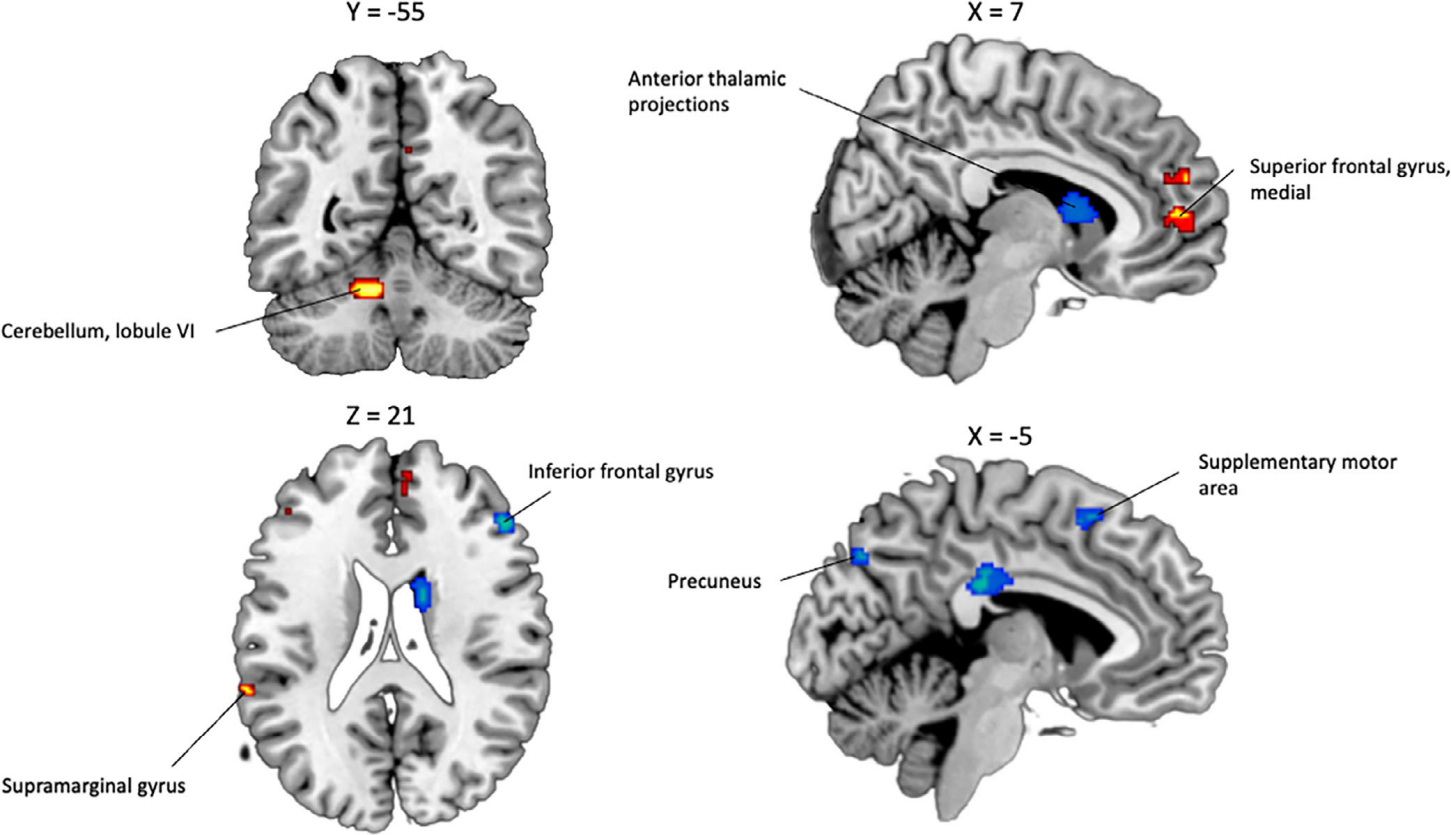
Significant brain functional activations and deactivations associated with reality‐monitoring (self > nonself) estimated by a whole‐brain meta‐analysis. The results are displayed based on the uncorrected threshold of *p* < .005 (minimum cluster size = 20 voxels) and overlaid on sagittal and axial sections of a normalized canonical template brain (ch2better) using MRIcron software. Coordinates are reported in Montreal Neurological Institute (MNI) space. The intensity color scale indicates Z‐score values (colors should be used for Figure 3 to print).

The SDM meta‐analysis revealed significant activations associated with reality‐monitoring (self > nonself) in the lobule VI of the left cerebellum, the right medial superior frontal gyrus (BA 10, amPFC) and the left supramarginal gyrus (BA 48) (see Table 2, Figure 3). Results also revealed significant deactivations in the right anterior thalamic projections, the left median cingulate gyrus (BA 23), the right inferior frontal gyrus (BA 45), the left precuneus (BA 7), the left caudate nucleus, the left supplementary motor area (BA 6), and the left fusiform gyrus (BA 37). Only the deactivation in the right anterior thalamic projections survived to FWE‐correction. This peak was associated with low between‐study heterogeneity (*I* ^2^ = 0.62%). Egger's test results and funnel plot observations suggested that none of results were driven by publication bias (*p* = .53) (see Supplementary Figure 1). Finally, no significant effect was observed between our results and moderators (age, quality of the study, see Supplementary Table 7). Robustness analyses indicated that these findings were consistent in most studies (see Supplementary Table 5).

**TABLE 2:** Significant activation/deactivation for the reality‐monitoring meta‐analysis (Self > nonself contrast).

Cluster description							
Macroanatomical label	Cytoarchitectonic label	Number of voxels	*p*‐Value	*I* ^2^ (%)	Egger test *p*‐value	MNI	SDM‐Z
*Activation*							
Left cerebellum, hemispheric lobule VI		135	.00009	8.3	.563	−14, −50, −26	3.720
Right superior frontal gyrus, medial, BA 10	Area p32	54	.00077	4.2	.211	6, 52, 6	3.167
Left supramarginal gyrus, BA 48/left superior temporal gyrus	Area PFcm	21	.00114	15.8	.519	−60, −40, 24	3.052
*Deactivation*							
Right anterior thalamic projections		285	.00011^a^	0.6	.922	10, 8, 8	−4.448
Left median cingulate, BA 23		135	.00030	3.6	.932	−2, −24, 30	−3.701
Right inferior frontal gyrus, triangular part	Area 45	40	.00076	1.4	.956	50, 32, 20	−3.430
Left precuneus, BA 7	Area 7P	26	.00040	12.4	.978	−8, −76, 40	−3.169
Left caudate nucleus		22	.00107	1.2	.563	−12, 2, 18	−3.351
Left supplementary motor area, BA 6	Area 6mr	23	.00153	2.9	.988	−6, 14, 56	−3.071

*Note*: Regional differences in activation are based on the uncorrected threshold of *p* < .005, minimal cluster >20. Coordinates are reported in the standardized MNI space.

Abbreviations: BA, Broadmann area; *I* ^2^, percentage of variance attributable to heterogeneity; MNI, Montreal Neurological Institute; SDM‐Z, seed‐based d mapping Z‐value.

^a^ Survived to the family‐wise error rate correction threshold of *p* < .05.

### 3.2 Brain responses associated with self‐monitoring

**FIGURE 4:**
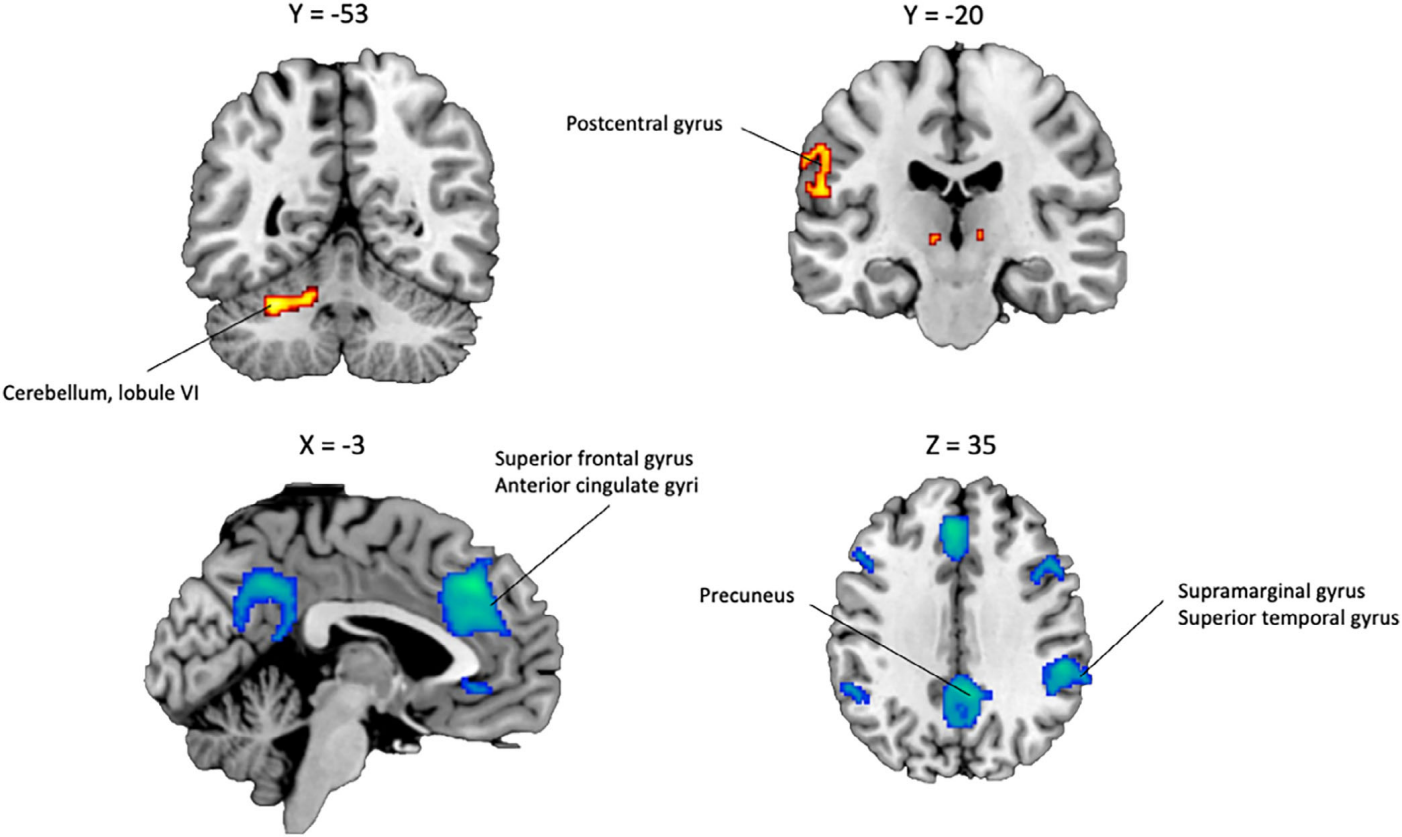
Significant brain functional activations and deactivations associated with self‐monitoring (self > nonself) estimated by a whole‐brain meta‐analysis. The results are displayed based on the uncorrected threshold of *p* < .005 (minimum cluster size = 20 voxels) and overlaid on sagittal and axial sections of a normalized canonical template brain (ch2better) using MRIcron software. Coordinates are reported in Montreal Neurological Institute (MNI) space. The intensity color scale indicates Z‐score values (colors should be used for Figure 4 to print).

The SDM meta‐analysis revealed significant activations associated with self‐monitoring (self > nonself) in the bilateral cerebellum, hemispheric lobule VI, the left supramarginal and postcentral gyrus (BA 48), the corpus callosum, the right supplementary motor area (BA 6), the right caudate nucleus, and the left thalamic projections (see Table 3). The results also revealed significant deactivations in the right supramarginal gyrus (BA 40), the right precuneus, the left anterior cingulate and medial superior frontal gyrus (BA 32), the left inferior parietal gyrus (BA 40), the right inferior frontal gyrus (BA 48), the left inferior frontal gyrus (BA 44), the right anterior cingulate and paracingulate gyri (BA 11), and the right middle temporal gyrus (BA 48) (see Figure 4). The activation in the left cerebellum, hemispheric lobule VI, and the left postcentral gyrus (BA 48) survived to FWE‐correction, as well as the deactivation in the right supramarginal gyrus (BA 40), the left anterior cingulate and medial superior frontal gyrus (BA 32), the right precuneus, and the left inferior parietal gyrus (BA 40).

**TABLE 3:** Significant activation/deactivation for the self‐monitoring meta‐analysis (Self > nonself contrast).

Cluster description							
Macroanatomical label	Cytoarchitectonic label	Number of voxels	*p*‐Value	*I* ^2^ (%)	Egger test *p*‐value	MNI	SDM‐Z
*Activation*							
Left cerebellum, hemispheric lobule VI		347	.00001^a^	6.7	.381	−28, −58, −26	4.169
Left postcentral gyrus, BA 48	Area OP1	288	.00017^a^	3.2	.364	−56, −18, 18	3.583
Corpus callosum	Area hPO1	175	.00010	5.5	.764	22, −76, 32	3.707
Right supplementary motor area, BA 6	Area 6d1	60	.00148	9.1	.511	16, 0, 62	2.971
Corpus callosum	Area hOc2	42	.00094	11.5	.574	20, −94, 10	3.108
*Deactivation*							
Right supramarginal gyrus, BA 40/right superior temporal gyrus, BA 22	Area hIP2	1229	<.00001^a^	3.3	.486	48, −42, 42	−5.223
Right precuneus		887	<.00001^a^	4.3	.137	4, −52, 40	−4.869
Left superior frontal gyrus, BA 32/left anterior cingulate gyri, BA 32	Area p32	844	<.00001^a^	5.6	.520	−4, 34, 38	−5.002
Left inferior parietal gyri, BA 40	Area hIP2	412	.00002^a^	5.8	.340	−44, −52, 52	−4.130
Right inferior frontal gyrus, opercular part, BA 48	Area 45	216	.00027	4.8	.279	48, 18, 30	−3.463
Left inferior frontal gyrus, triangular part, BA 48	Area 45	102	.00044	20.3	.211	−52, 22, 30	−3.327
Right anterior cingulate/paracingulate gyri, BA 11	Area p24ab	58	.00125	7.9	.468	4, 34, −6	−3.126
Right middle temporal gyrus, BA 48		52	.00125	10.2	.648	50, −16, −10	−3.296
Right middle temporal gyrus, BA 21		21	.00305	15.6	.301	58, −44, −4	−3.024

*Note*: Regional differences in activation are based on the uncorrected threshold of *p* < .005, minimal cluster >20. Coordinates are reported in the standardized MNI space.

Abbreviations: BA, Broadmann area; *I* ^2^, percentage of variance attributable to heterogeneity; MNI, Montreal Neurological Institute; SDM‐Z, seed‐based d mapping Z‐value.

^a^ Survived to the family‐wise error rate correction threshold of *p* < .05.

Low between‐study heterogeneity has been associated with each significant peak (*I* ^2^ = 3.23–6.71%). Egger's test results and funnel plot observations suggested that none of results were driven by publication bias (*p* = .14–.52) (see Supplementary Figure 2). No significant effect was observed between our results and moderators (age and quality of the study, see Supplementary Table 8). Robustness analyses indicated that these findings were consistent in most studies (see Supplementary Table 6).

### 3.3 Overlap between reality‐monitoring and self‐monitoring brain reactivity

**FIGURE 5:**
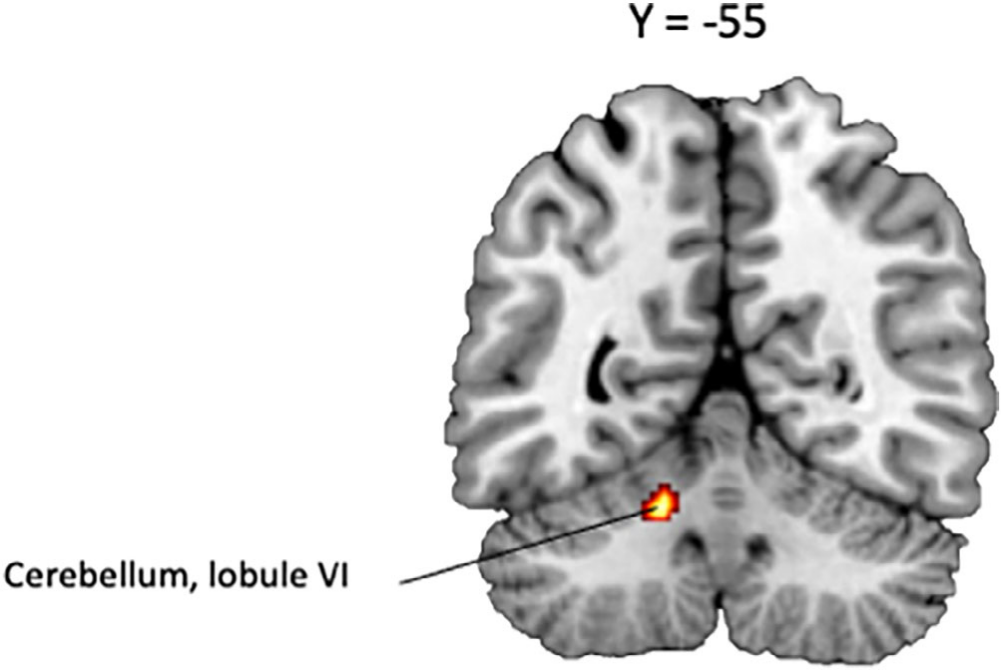
Significant brain functional activations reflecting the overlap between reality‐monitoring and self‐monitoring whole‐brain meta‐analyses. The results are displayed based on the uncorrected threshold of *p* < .005 (minimum cluster size = 20 voxels) and overlaid on sagittal and axial sections of a normalized canonical template brain (ch2better) using MRIcron software. Coordinates are reported in Montreal Neurological Institute (MNI) space. The intensity color scale indicates Z‐score values (colors should be used for Figure 5 to print).

We finally performed a conjunction analysis to identify the overlapping brain regions between both self‐monitoring and reality‐monitoring meta‐analytic statistical maps. Results revealed significant activation of the left cerebellum, lobule VI in the self > nonself contrast in both paradigms of self‐agency (see Table 4, Figure 5). This finding was not detectable after FWE‐correction. The coordinates of this part of the cerebellum were used to extract a mask from the two main analyses. Activation in this region was associated with similar effect size and low heterogeneity in both self‐monitoring and reality‐monitoring (Hedge's *g* = 0.36 and 0.40, *I* ^2^ = 1.08 and 19.17%, respectively).

**TABLE 4:** Significant activation/deactivation for the conjunction meta‐analysis between reality‐monitoring and self‐monitoring.

Macroanatomical label	Number of voxels	*p*‐Value	MNI	SDM‐Z
*Self > nonself* (*activation*)				
Left cerebellum, hemispheric lobule VI	53	.00046	−14, −52, −26	3.310
*Self < nonself* (*deactivation*)				
No significant results				

*Note*: Regional differences in activation are based on the uncorrected threshold of *p* < .005, minimal cluster >20. Coordinates are reported in the standardized MNI space. Range of quality score: 0–1.

Abbreviations: MNI, Montreal Neurological Institute; SDM‐Z, seed‐based d mapping Z‐value.

### 3.4 Supplemental analysis results: Comparison between paradigms

**FIGURE 6:**
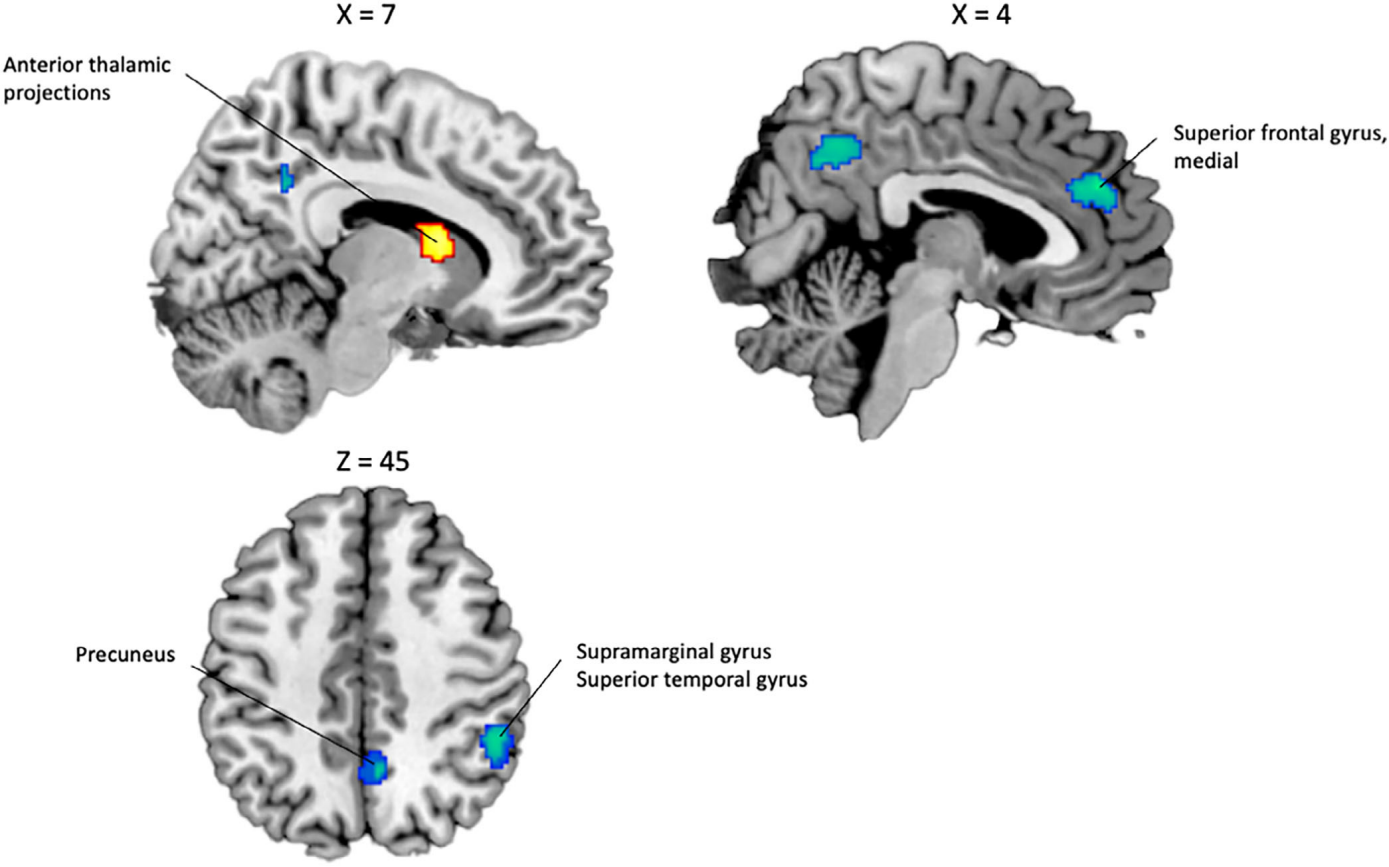
Significant brain functional activations reflecting the difference between reality‐monitoring and self‐monitoring whole‐brain meta‐analyses (Self‐monitoring > Reality‐monitoring contrast). The results are displayed based on the uncorrected threshold of *p* < .005 (minimum cluster size = 20 voxels) and overlaid on sagittal and axial sections of a normalized canonical template brain (ch2better) using MRIcron software. Coordinates are reported in Montreal Neurological Institute (MNI) space. The intensity color scale indicates Z‐score values (colors should be used for Figure 6 to print).

The direct comparison between reality‐monitoring and self‐monitoring studies reports significantly more deactivation in the right anterior thalamic projections in reality‐monitoring studies compared to self‐monitoring studies. The contrast analysis also revealed significantly more deactivation in the right supramarginal gyrus, the right anterior cingulate gyri and the right precuneus in self‐monitoring studies compared to reality‐monitoring studies (see Table 5, Figure 6).

**TABLE 5:** Significant results of the comparison between reality‐monitoring and self‐monitoring studies (Self‐monitoring > Reality‐monitoring contrast).

Macroanatomical label	Number of voxels	*p*‐Value	MNI	SDM‐Z
Right anterior thalamic projections	138	.00006	12, 6, 12	3.852
Right supramarginal gyrus, BA 40	319	.00011	52, −44, 42	−3.697
Right anterior cingulate/paracingulate gyri, BA 32	227	.00006	4, 42, 26	−3.833
Right precuneus	196	.00003	6, −58, 36	−4.048

*Note*: Regional differences in activation are based on the uncorrected threshold of *p* < .005, minimal cluster >20. Coordinates are reported in the standardized MNI space. None of the identified clusters survived to the family‐wise error rate correction threshold of *p* < .05.

Abbreviations: MNI, Montreal Neurological Institute; SDM‐Z, seed‐based d mapping Z‐value.

### 3.5 Supplemental analysis results: Controlling for confounders

The study quality had no significant influence on the functional results in either self‐monitoring or reality‐monitoring. Higher age was significantly associated with activation in the left inferior frontal gyrus (BA 44) and the right supplementary area during the self‐monitoring paradigm (see Supplementary Table 8). None of these brain regions overlapped with areas that survived FWE‐correction. Finally, age had no significant influence on the reality‐monitoring results (see Supplementary Table 7).

## 4 DISCUSSION

While self‐ and reality‐monitoring conceptually overlaps in the sense that they both involve distinguishing self from nonself origins of information, the two concepts remain largely separate cognitive fields; moreover, their common brain substrates have received relatively little attention. Using a coordinate‐based meta‐analysis, we compiled and analyzed results from imaging studies investigating the brain correlates of either reality‐monitoring and self‐monitoring and examined their overlapping neural responses. We identified specific brain regions involved in reality‐monitoring and confirmed the central role played by the bilateral IPL in self‐monitoring (Seghezzi et al., 2019; Sperduti et al., 2011). Importantly, our findings suggest consistent activation of the lobule VI of the left cerebellum in both reality‐monitoring and self‐monitoring.

### 4.1 Brain areas involved in reality‐monitoring

Our meta‐analysis revealed that the right amPFC is consistently activated during reality‐monitoring. Its activation has been shown using a liberal statistical threshold (i.e., *p* < .005, uncorrected, minimal cluster size >20) but not when using a conservative threshold (i.e., *p* < .05 FWE‐corrected). That being said, this activation is highly consistent with a large corpus of studies showing that the amPFC exhibits differential activity during the retrieval of internally vs. externally generated information using a ROI approach (for review, Simons et al., 2017). All the studies that were bound to the scope of an a priori ROI were excluded from the present meta‐analysis. However, these studies demonstrated that the amPFC modulates its activity during a more diverse range of reality‐monitoring tasks that those we included, using verbal items, but also faces and objects (Dobbins & Wagner, 2005; Kensinger & Schacter, 2006; Simons, Gilbert, et al., 2005; Simons, Owen, et al., 2005). Our findings are also in line with interventional studies using neurofeedback or brain stimulation that have shown the causal involvement of amPFC in reality‐monitoring (Garrison et al., 2021; Subramaniam et al., 2020). Here, the activation of the amPFC during reality‐monitoring but not self‐monitoring suggests its specific role in attributing the source of memories through the distinction between the retrieval of their internal and external features. These findings are consistent with the hypothesis that the amPFC plays a higher‐order role of evaluation of low‐level sensory signals and cognitive control aspects of perception and imagination in order to make a source attribution (Dijkstra et al., 2022). At the structural level, the reduction in length in the paracingulate sulcus (PCS), a tertiary sulcus surrounding the ACC, is associated with reduced reality‐monitoring performance (Buda et al., 2011; Fornito et al., 2008). The pathological implication of this structural variability has been shown in patients with schizophrenia, for whom the reduction in PCS length is associated with hallucinations (Garrison et al., 2015) and deficits of reality monitoring (Perret et al., 2021). If the relationship between brain morphometry and functional activity remains unclear, the cortical folding may influence the functional involvement of the amPFC and ACC during reality‐monitoring. Our meta‐analysis also identified a specific activation in the left SMG. However, this activation was associated with 16% of heterogeneity, only concerned a small number of voxels and did not survive to FWE‐correction. Finally, the only cluster surviving to FWE‐correction has peaks in the right anterior thalamic projections. Its specific role in the self versus nonself distinction should be further explored. Given the crucial projection from the anterior thalamus to the anterior cingulate cortex within the Papez circuit supporting neural substrates of memory (Jankowski et al., 2013; Papez, 1937), future studies should pay particular attention to their functional connectivity during reality‐monitoring.

### 4.2 Brain areas involved in self‐monitoring

Concerning self‐monitoring, we identified activations in the left cerebellum and postcentral gyrus and deactivations in the right supramarginal gyrus and the left anterior cingulate and the medial superior frontal gyrus. The deactivation of the right SMG corroborates the findings of Seghezzi et al. (2019) and Sperduti et al. (2011). Substantiating its pivotal role in self versus nonself distinction, hyperactivity of the right IPL in response to self‐generated events is correlated with symptoms that include delusion of alien control, insertion‐of‐thought experiences, and hallucinations in schizophrenia patients (Jardri, Pouchet, et al., 2011; Spence et al., 1997). Furthermore, a distinct sulcal pattern distribution of the Sylvian fissure, a sulcus surrounding the right IPL, has been observed in patients with schizophrenia who misattribute their hallucinations to an external source compared to patients who recognize that they originate from their own thoughts (Plaze et al., 2015). One can assume that such anatomical variability in pathological condition gives an indication as to the functional role of the right IPL in disentangling the origin of online information. But how would the deactivation of the right IPL participate in self‐monitoring? Interestingly, the right IPL is involved in various tasks, such as go/no go, false‐belief reasoning, and theory of mind, which also require online comparison between internal predictions and external perceived events (Decety & Lamm, 2007; Rothmayr et al., 2011). As a core region of the ventral frontoparietal network, the right SMG is indeed engaged in attention reorienting from an internal model to externally directed information in the context of a violation of expectations (Corbetta et al., 2008). Moreover, as a part of the secondary somatosensory cortex, the right SMG receives strong connections from sensory and motor areas such as the left postcentral gyrus and has specifically been involved in attentional modulation of somatosensory stimuli (Chen et al., 2008; Fujiwara et al., 2002; Hämäläinen et al., 2002). Hence, the right SMG deactivation during self‐monitoring could reflect the sensory dampening observed in the context of self‐generated action and lead to maintaining or redirecting attention toward internally generated stimuli. Our meta‐analysis also confirms the findings of Sperduti et al. and Seghezzi et al. on the key role of the left IPL for external agency during self‐monitoring. This region has previously been associated with the detection of incongruent feedback during action execution (Balslev et al., 2006). In schizophrenia patients, hyperactivation of the left IPL during self‐monitoring is associated with false signaling of incongruence and passivity symptoms, characterizing the experience of believing that one's thoughts or actions are controlled by an external agent (Frith, 2005; Schnell et al., 2008). One could then assume that the left IPL deactivation reflects the absence of conflict in the event of congruence between the predicted and actual feedback of self‐generated action. Our cytoarchitectonic analysis further specified the localization of this deactivation in the hIP2 region of the left IPL, displaying strong functional connectivity with the right SMG and the left superior frontal gyrus (Uddin et al., 2010), which also deactivate during the self versus nonself distinction. The latter is also involved in conflictual decision making: the left superior frontal gyrus and ACC are specifically associated with confusion between imagined and perceived pictures (Gonsalves et al., 2004; Stephan‐Otto, Siddi, Senior, Cuevas‐Esteban, et al., 2017) and their disruption is associated with deficits in error‐monitoring in schizophrenia patients (Alain et al., 2002; Mathalon et al., 2002). In the context of self‐agency, this suggests that the left superior frontal gyrus and ACC act conjointly with the left IPL in monitoring the conflicts between predicted and observed stimuli.

### 4.3 Functional convergence between reality‐monitoring and self‐monitoring: Is the lobule VI of the cerebellum a key structure for self‐agency?

The conjunction analysis between reality‐monitoring and self‐monitoring has revealed robust common activation of the left lobule VI of the cerebellum. This activation further corroborates the *cerebellar forward model*, indicating that self‐generated productions lead the cerebellum to generate sensory predictions (Pinheiro et al., 2020; Sokolov et al., 2017). After finding a selective response of the lobule VI when tactile stimuli were self‐produced, Blakemore et al. assumed for the first time that the cerebellum receives an efference copy of motor commands to build the prediction of their somatosensory consequences (Blakemore et al., 1998). The generation of the expected sensory outcome has been hypothesized to then reduce the activity of the implicated sensory areas. For instance, amplitude reduction of the N1 event‐related response and reduced BOLD activity of the auditory cortex after voice onset seem to reflect a match between self‐generated motor‐to‐auditory prediction and actual sensory feedback (Baess et al., 2009, 2011; Christoffels et al., 2007; Numminen et al., 1999; Sato & Shiller, 2018). Such a role of cerebrocerebellar pathways has been demonstrated by studies reporting patients with cerebellar lesions to not display any N100 suppression after a self‐generated sound (Knolle et al., 2012, 2013). In the same way, actions with predictable visual consequences are associated with BOLD suppression in visual cortices and greater cerebellar‐visual cortex connectivity than actions with unpredictable visual consequences (Straube et al., 2017). Furthermore, several fMRI studies highlighted the role of the cerebellum during language prediction (Lesage et al., 2017; Moberget et al., 2014) and used neurostimulation to demonstrate causality between cerebellum activation and the ability to anticipate words in a sentence (D'Mello et al., 2017; Lesage et al., 2012; Miall et al., 2016). In a subsequent study, Blakemore et al. also found the lobule VI to modulate its activity when increasing the delay between a hand's movement and the resulting tactile stimulation of a passive hand, suggesting that this region should constantly compare expected and actual sensory feedback to detect potential discrepancies (Blakemore et al., 2001). In response to mismatches, an error signal from the cerebellum would update the *forward model* by reducing the sensory suppression of the implicated sensory areas (Pinheiro et al., 2020). If activation of lobule VI of the cerebellum during online self‐agency is highly coherent with the cerebellum forward model, our results suggest that this region reactivates when remembering the self‐provenance of information during the reality‐monitoring retrieval phase. Previous studies implicated the cerebellum in both the encoding and retrieval aspects of episodic memory (i.e., the ability to recollect a specific personal experience, including the context) and in the acquisition and retention of motor memories (Herzfeld et al., 2014) using plasticity mechanisms (Andreasen et al., 1999; D'Angelo, 2014; Fliessbach et al., 2007; Fossati et al., 2004; Hirano, 2013; Ito, 2001). The cerebellum has been more specifically identified as a part of a neural network activated during source memory relative to object memory (Hawco et al., 2015), and lesions in this structure are associated with repeated misattributions between the self and the external origin of a memory (Tamagni et al., 2010). Based on its overlapping activation in self‐monitoring and reality‐monitoring, we hypothesized that the cerebellum plays a key role in the feed‐forward model and as a “cognitive cue” to identify the self‐origin of stored information.

### 4.4 Integrating the cerebellar forward model and the reality‐monitoring framework

The feed‐forward model accounting for the recognition of self‐generated productions proposes that the outgoing motor signal is accompanied by a replicate called the efference copy, and the integration of this replicate results in building a prediction of the sensory feedback. This prediction minimizes the sensory perception of our own actions or speech. In addition, a constant comparison between the prediction and the actual sensory input would allow the detection of potential discrepancies to update the forward model. What would be the neurobiological substrates of such a model? First, our results support the claim that the lobule VI of the cerebellum is a pivotal neural locus for recognizing self‐produced behaviors. According to this view, the cerebellum might integrate the efference copy of self‐productions to generate an expectation of sensory feedback, which would then transit by cerebello‐cortical connections to prepare the sensory areas for incoming sensory feedback. This sensory attenuation would be underpinned by the deactivation of somatosensory regions such as the left postcentral gyrus and the right SMG. In this way, the connectivity between lobule VI of the cerebellum and the right SMG has been suggested to support somatosensory attenuation in the context of self‐generated action (Kilteni & Ehrsson, 2020). Moreover, deactivation in the right TPJ would in turn lead to alleviate the accuracy of future expectations by maintaining or reallocating attention to internally generated stimuli. In the event of a mismatch between the expected and actual sensory feedback, an error signal would transit again from the cerebellum to the primary sensory cortices and motor areas to reduce the sensory attenuation and update the motor command, respectively. The difference between expected and actual sensory feedback would then be propagated to other hierarchical levels, such as the left IPL and ACC, involved in error processing and contributing to altered sense of agency. Within the *reality‐monitoring framework*, our meta‐analysis finally advocates for a reactivation of the lobule VI cerebellum during the retrieval phase of reality‐monitoring to reinstate the encoding context in collaboration with the amPFC. The amPFC would then integrate and evaluate the retrieved markers of the cognitive operations associated with thought, speech, and other actions to attribute its source.

Several limitations of this study should be acknowledged. First, even with a total of 172 individuals, we could only include 9 reality‐monitoring studies. Most of the studies mentioned in the Simons et al.'s review (Simons et al., 2017) have not been included in this quantitative meta‐analysis. Several of these studies have only reported ROI‐based analyses in the amPFC (Brandt et al., 2014; Simons et al., 2006, 2008; Vinogradov et al., 2008). The inclusion of experiments from different original search coverage would inflate the significance for the amPFC (Müller et al., 2018). Another portion of these studies reported the involvement of the amPFC in reality‐monitoring using heterogeneous contrasts that we excluded from the present meta‐analysis (e.g., misattributions of the source vs. correct attribution (Kensinger & Schacter, 2006), correct recognition of the source status vs. baseline (Simons et al., 2008)). Of note, Simons et al.'s paper is not a systematic review and most of the studies of reality‐monitoring in healthy subjects using fMRI that we included in the present meta‐analysis are not mentioned in their review. Second, due to the number of included studies, we were not able to subcategorize the experiments according to the modality of the stimulus (i.e., action, imagery, verbal tasks). Regarding the amPFC, previous studies adopting an ROI approach have shown a similar activation of the amPFC in imagery and verbal studies, suggesting that this region is involved in reality‐monitoring regardless of the modality. In the self‐monitoring meta‐analysis, only two studies used verbal tasks (Jardri et al., 2007; Jardri, Pins, et al., 2011). However, heterogeneity tests did not reveal any significant between‐study variance that could have indicated a verbal versus action difference. Nonetheless, comparing subgroups according to the stimulus dimension is certainly the most thorough way of controlling this potential confounder. Currently, the complete lack of whole‐brain fMRI reality‐monitoring contrasts using action stimuli and the small number of neuroimaging studies using verbal self‐monitoring contrasts prevent us from employing this kind of rigorous standard. A third limitation of the current study is the uncertainty about the inclusion of the cerebellum in whole‐brain analyses. Out of the 16 included studies, 4 did not reveal activation in this structure or specify whether their whole‐brain analysis covered the structure (Lundstrom et al., 2003; Renes et al., 2015; Subramaniam et al., 2012; Takahashi et al., 2002). Consequently, for these four studies, the lack of signal in the cerebellum may be considered a potential false negative. However, our meta‐analysis showed that the most substantial and consistent activation in the cerebellum occurred in response to self‐generated information; therefore, the only risk of bias might be a slight underestimation of the effect size. Nevertheless, further studies are needed to investigate self‐agency by systematically including the cerebellum in their whole‐brain coverage, and these works should specify whether the structure is included in the analyses. Fourth, we reported results with a statistical threshold of *p* < .005 (uncorrected, minimal cluster size >20). Although SDM developers demonstrated that the liberal threshold of *p* < .005 optimally balances sensitivity and specificity (Radua, Mataix‐Cols, et al., 2012) and this threshold has been mostly used in meta‐analyses of neuroimaging studies, it remains an approximation of the corrected results. When using a more conservative threshold of *p* < .05 (FWE‐corrected), the amPFC activation did not survive in the reality‐monitoring meta‐analysis and the conjunction meta‐analysis yielded no significant results. This could be due to several reasons: (a) even with a total number of 172 subjects included in the reality‐monitoring meta‐analysis, only nine studies were included, (b) the heterogeneity between verbal reality‐monitoring studies and action self‐monitoring studies could have reduced our ability to observe results surviving conservative thresholding, and (c) coordinate‐based meta‐analyses are susceptible to threshold bias (we were not able to ask for unthresholded maps because most of the included studies were published more than 10 years ago). We reported results with both thresholded and unthresholded *p*‐values to move beyond p‐value and discussed the amPFC activation in the light of the converging evidence from numerous ROI studies showing its consistent involvement in reality‐monitoring. Concerning the conjunction meta‐analysis, we supplemented the unthresholded *p*‐values by extracting masks that allowed us to report similar moderate effect‐sizes in the cerebellum peak for both self‐monitoring and reality‐monitoring meta‐analyses. A last caveat of this study is the inclusion of slightly heterogeneous contrasts in the reality‐monitoring meta‐analysis. Seven over nine studies reported a self versus nonself contrast regardless the correct identification of the source. Two studies (Stephan‐Otto, Siddi, Senior, Muñoz‐Samons, et al., 2017; Takahashi et al., 2002), however, reported the contrast between correctly remembered self‐generated items and correctly remembered nonself‐generated items. We tested the robustness of our results by replicating our meta‐analysis while excluding these studies and showed no difference with the original meta‐analysis.

## 5 CONCLUSIONS

Based on the common cognitive substrate of reality‐ and self‐monitoring, we adopted a metanalytic approach to investigate the brain regions that are involved in either of these two paradigms and performed conjunction analysis to highlight their overlaps. Our results suggest that the lobule VI of the cerebellum is consistently engaged in both reality‐ and self‐monitoring. This finding is highly consistent with the *cerebellar forward model*, in which the cerebellum plays a key role in generating the predicted feedback of our own actions and producing an error signal in the event of a mismatch with the actual sensory feedback. During self‐monitoring, the cerebellum would act together with cerebral regions including the right TPJ and left IPL and ACC. When remembering the self‐origin of information at the retrieval phase of reality‐monitoring, the cerebellum would reactivate within a set of brain regions including the right amPFC and anterior thalamic projections. Because the exact functions of these structures remain highly speculative, our results set the rationale for future imaging and brain stimulation studies that may explore their contribution to self‐agency. Finally, this study has far‐reaching implications for a better understanding of altered reality‐monitoring in the context of schizophrenia, in which patients experience a severe blurring of the self/nonself‐distinction and confusion between self‐generated stimuli and those they perceive from the environment (Brookwell et al., 2013; Waters et al., 2012).

## AUTHOR CONTRIBUTIONS

Layla Lavallé: Conceptualization, methodology, formal analysis, visualization, writing – original draft, writing – review and editing. Marine Mondino: Conceptualization, methodology, writing – review and editing. Jérôme Brunelin: Conceptualization, methodology, writing – review and editing. Frédéric Haesebaert: Writing – review and editing. Renaud Jardri: Writing – review and editing.

## CONFLICT OF INTEREST

The authors declare no conflict of interest.

## Supporting information


**DATA S1** Supporting Information

## Data Availability

All data used in this study were obtained from original publications. Aggregated data are shared on the Open Science Framework (https://osf.io/7xm9t/?view_only=c92372b173c742f98fd2d54b3acee328). Meta‐analyses that we used in this study were achieved via the software SDM‐PSI (https://www.sdmproject.com/). The unthresholded maps from meta‐analyses are publicly available at: https://neurovault.org/collections/12882/.
