# Supplementary material for: The neural signature of reality‐monitoring: A meta‐analysis of functional neuroimaging studies
Source: Hum Brain Mapp. 2023 May 29;44(11):4372–89. doi: 10.1002/hbm.26387 (PMC10318245; doi:10.1002/hbm.26387)
Supplement: Supplementary file 1 — DATA S1 Supporting Information [file HBM-44-4372-s001.docx]

**Neural Signature of Self-Agency: A Meta-Analysis of Functional Neuroimaging Studies**

**Supplementary Information**

**Authors:**

Layla LAVALLÉ ^1,2^, Frédéric HAESEBAERT ^1,2^, Renaud JARDRI ^3^, Jérôme BRUNELIN ^1,2^, Marine MONDINO ^1,2*^

**Affiliations:**

1. Université Claude Bernard Lyon 1, CNRS, INSERM, Centre de Recherche en Neurosciences de Lyon CRNL U1028 UMR5292, PSYR2, Bron F-69500, France
2. CH le Vinatier, F-69500 Bron, France
3. Université de Lille, INSERM U-1172, Lille Neurosciences & Cognition, Plasticity & Subjectivity Team, CHU Lille, France

***Corresponding author:** [marine.mondino@ch-le-vinatier.fr](mailto:marine.mondino@ch-le-vinatier.fr)

**Supplementary Figures**

**Supplementary Figure 1:** Funnel plots of the activation and deactivation effect-sizes in the reality-monitoring meta-analysis (A: Left cerebellum, Eggers’ test p-value = 0.563, B: Right superior frontal gyrus, Eggers’ test p-value = 0.211, C: Right anterior thalamic projections, Eggers’ test p-value = 0.922). The horizontal axis represents the effect-size. The vertical axis represents the standard error.
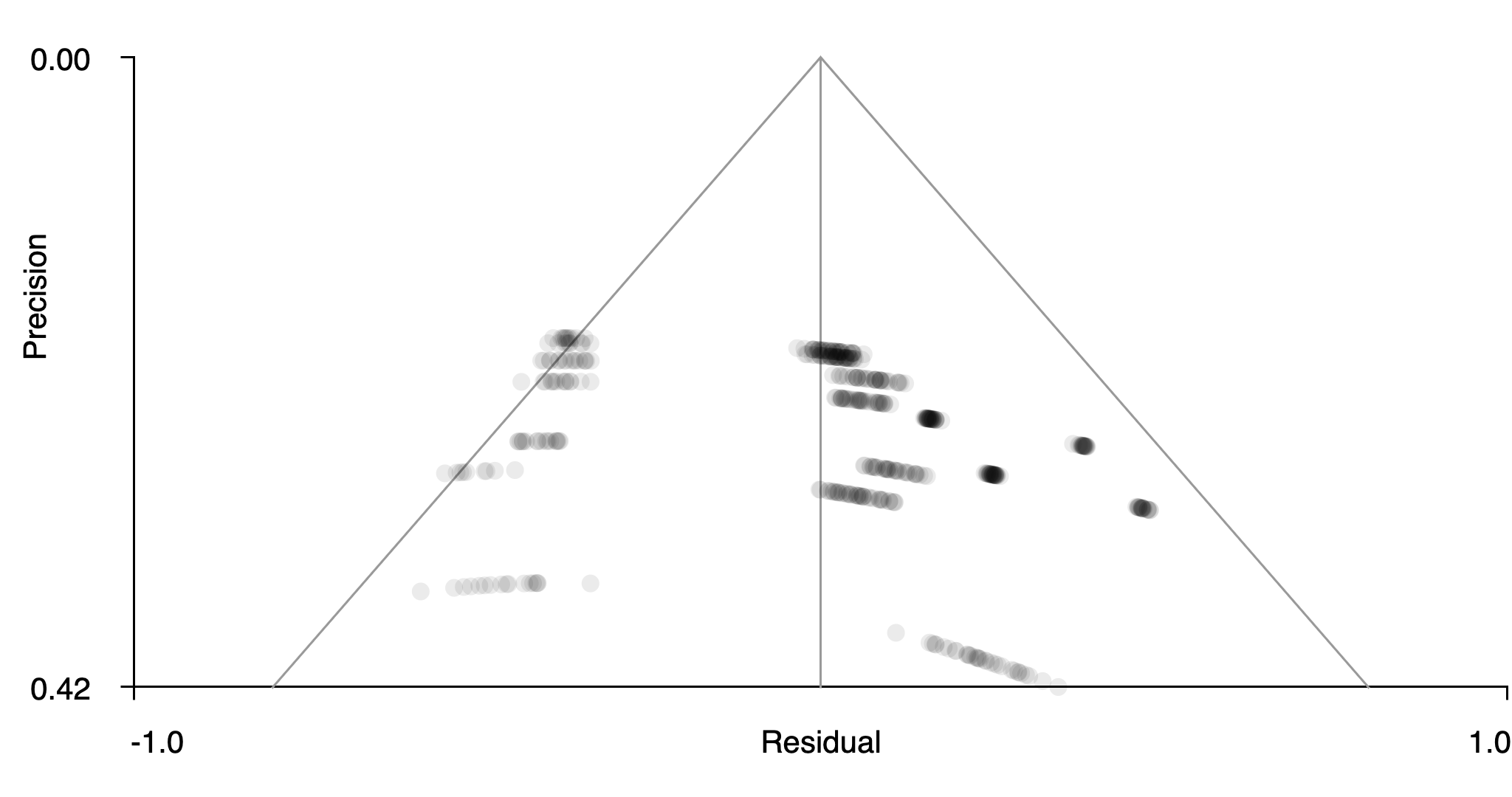


A


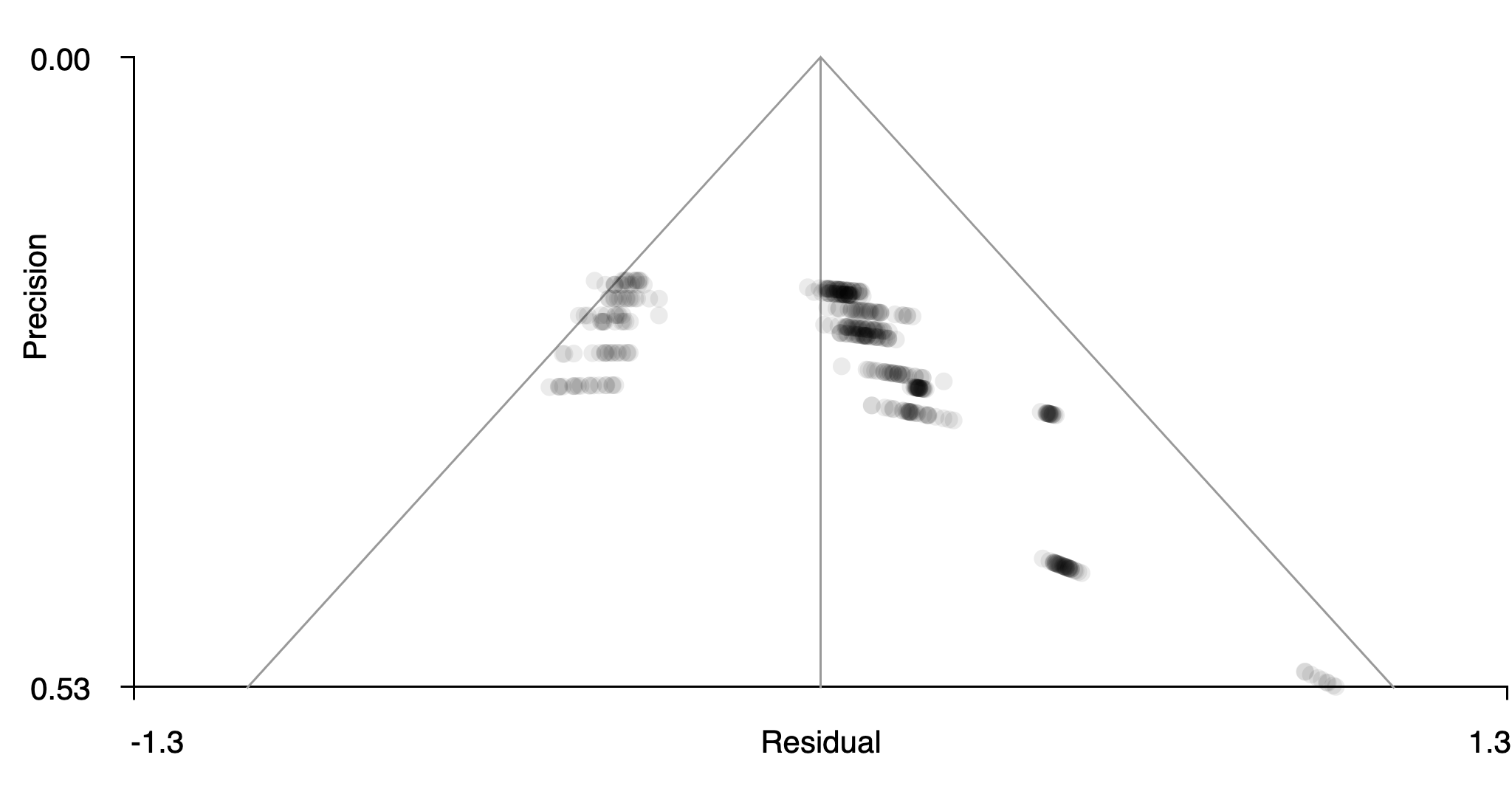


B


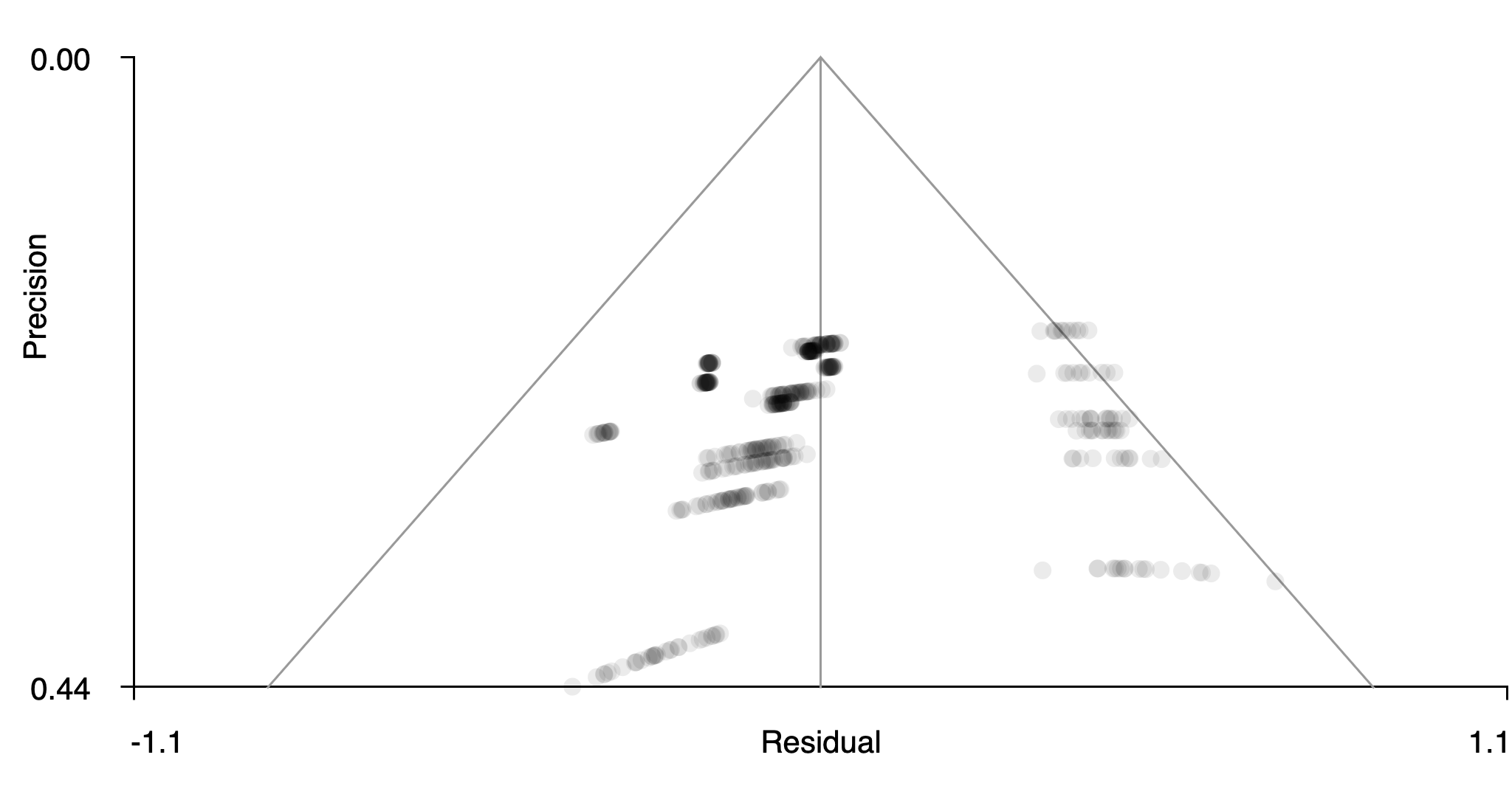


C

**Supplementary Figure 2:** Funnel plots of the activations and deactivations effect-sizes in the self-monitoring meta-analysis (A: Left cerebellum, Eggers’ test p-value = 0.381, B: Left postcentral gyrus, Eggers’ test p-value = 0.364, C: Right supramarginal gyrus / Right superior temporal gyrus, Eggers’ test p-value = 0.486, D: Right precuneus, Eggers’ test p-value = 0.137, E: Left superior frontal gyrus / Left anterior cingulate gyri, Eggers’ test p-value = 0.520, F: Left inferior parietal gyri, Eggers’ test p-value = 0.340). The horizontal axis represents the effect-size. The vertical axis represents the standard error.


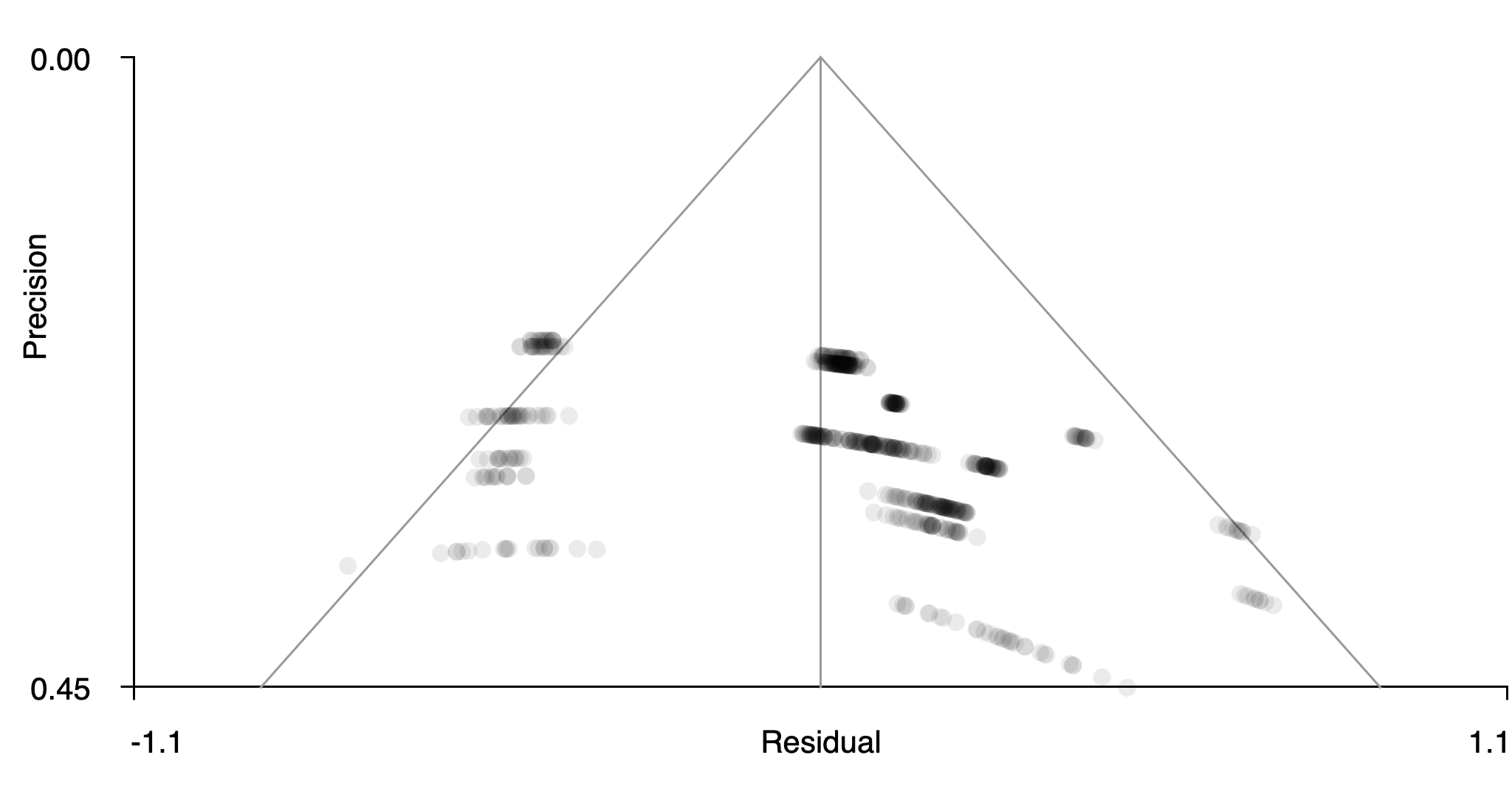


A


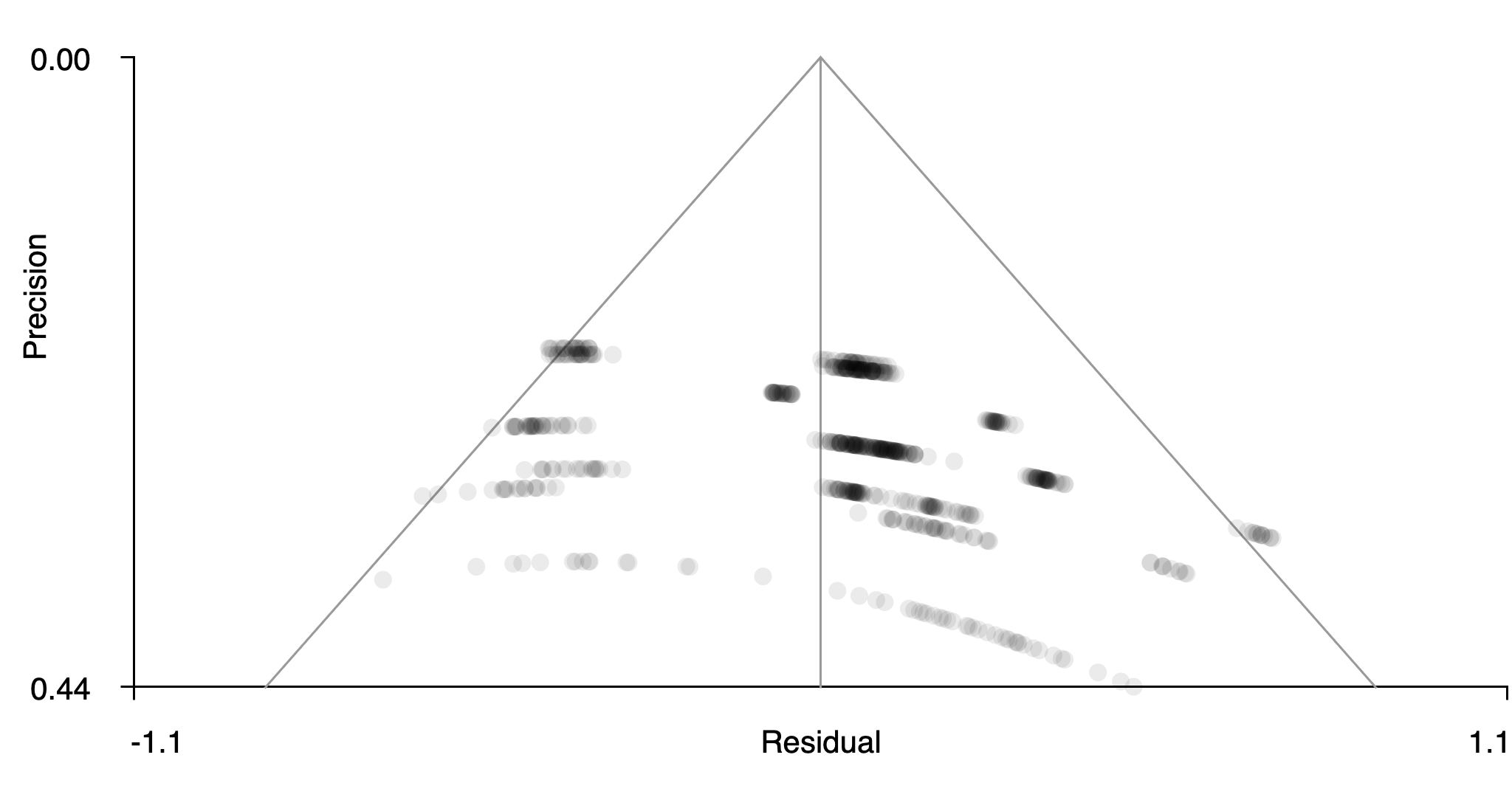


B


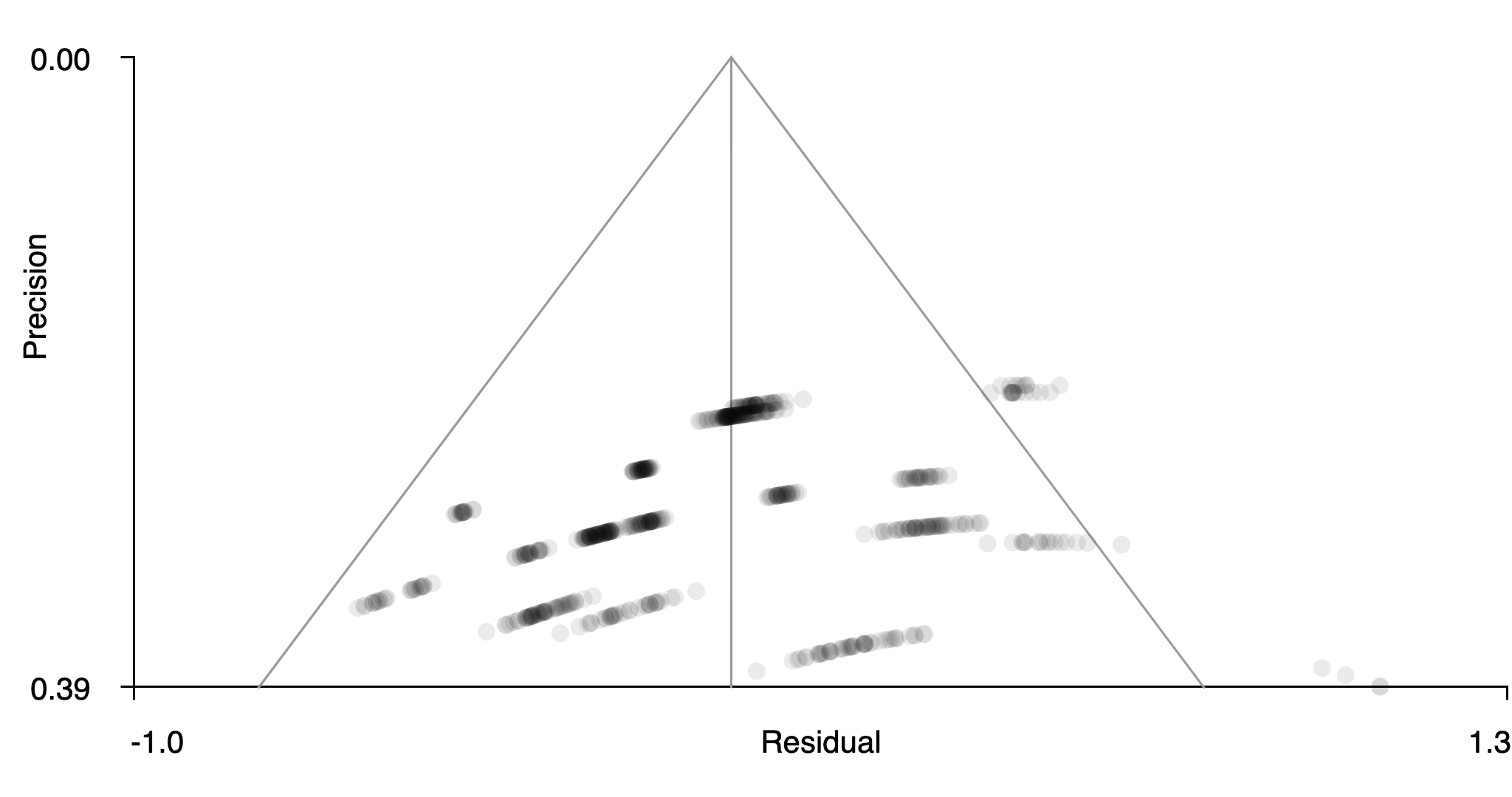


C


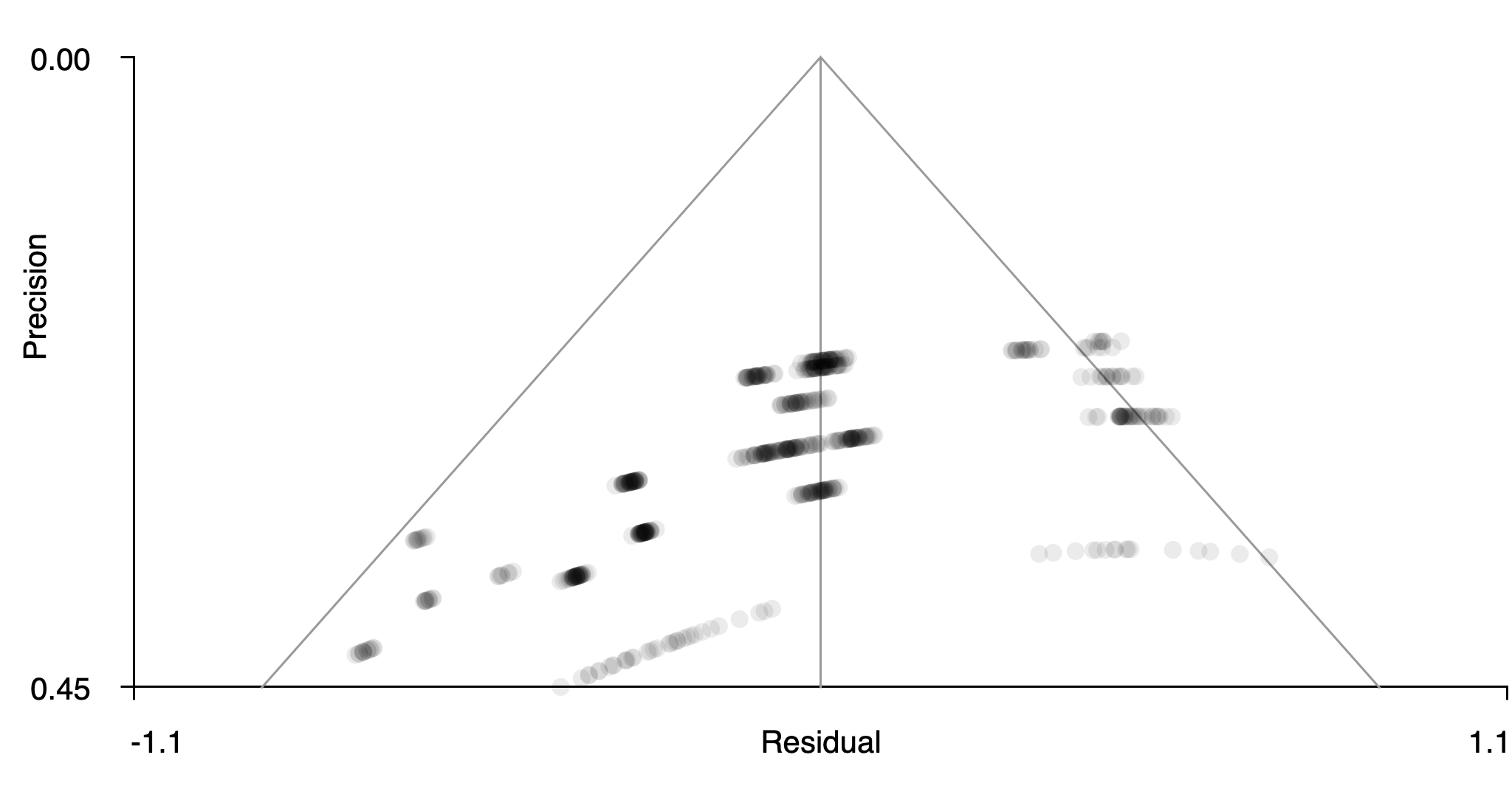


D

**
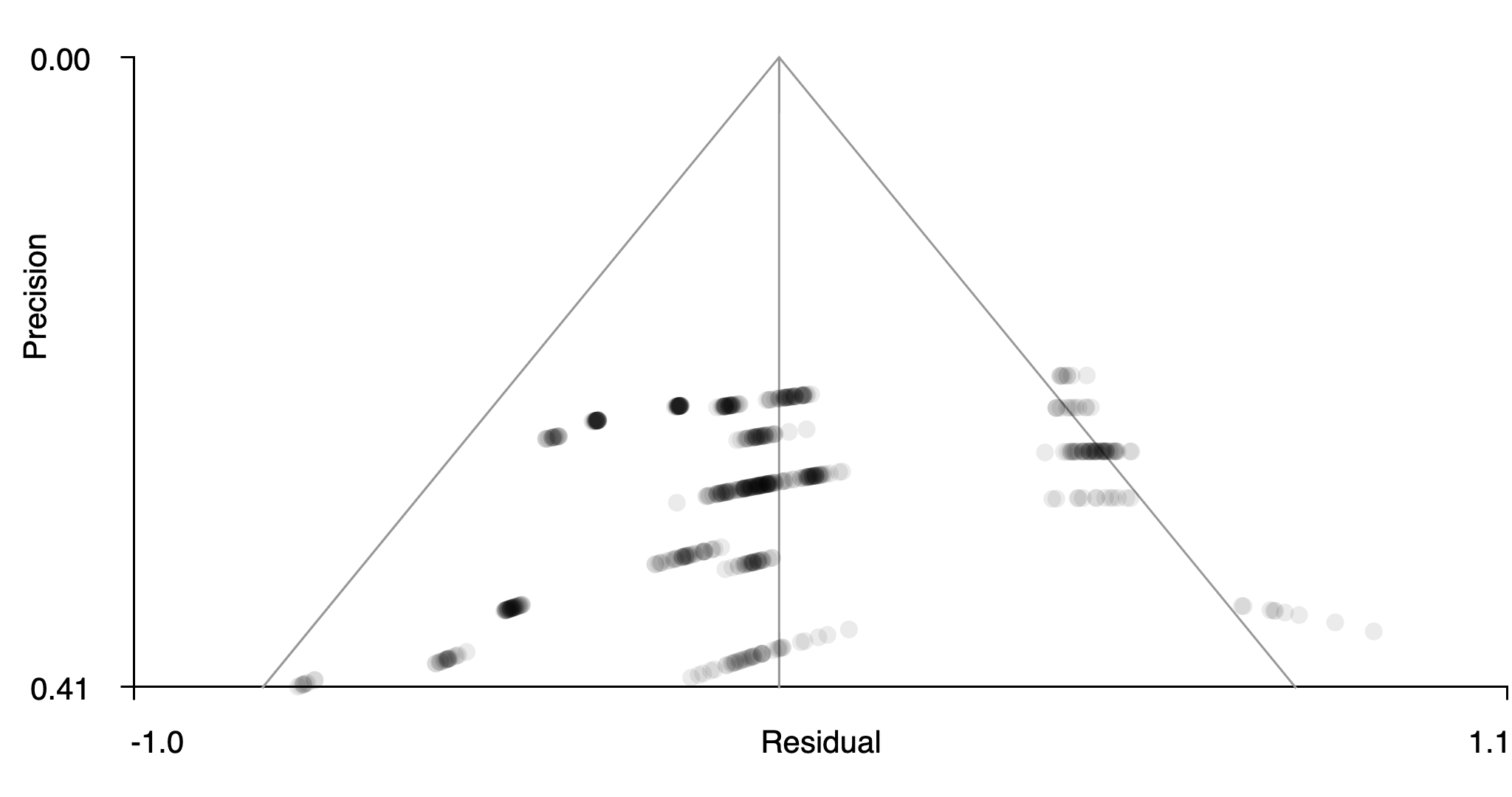
**

E

**
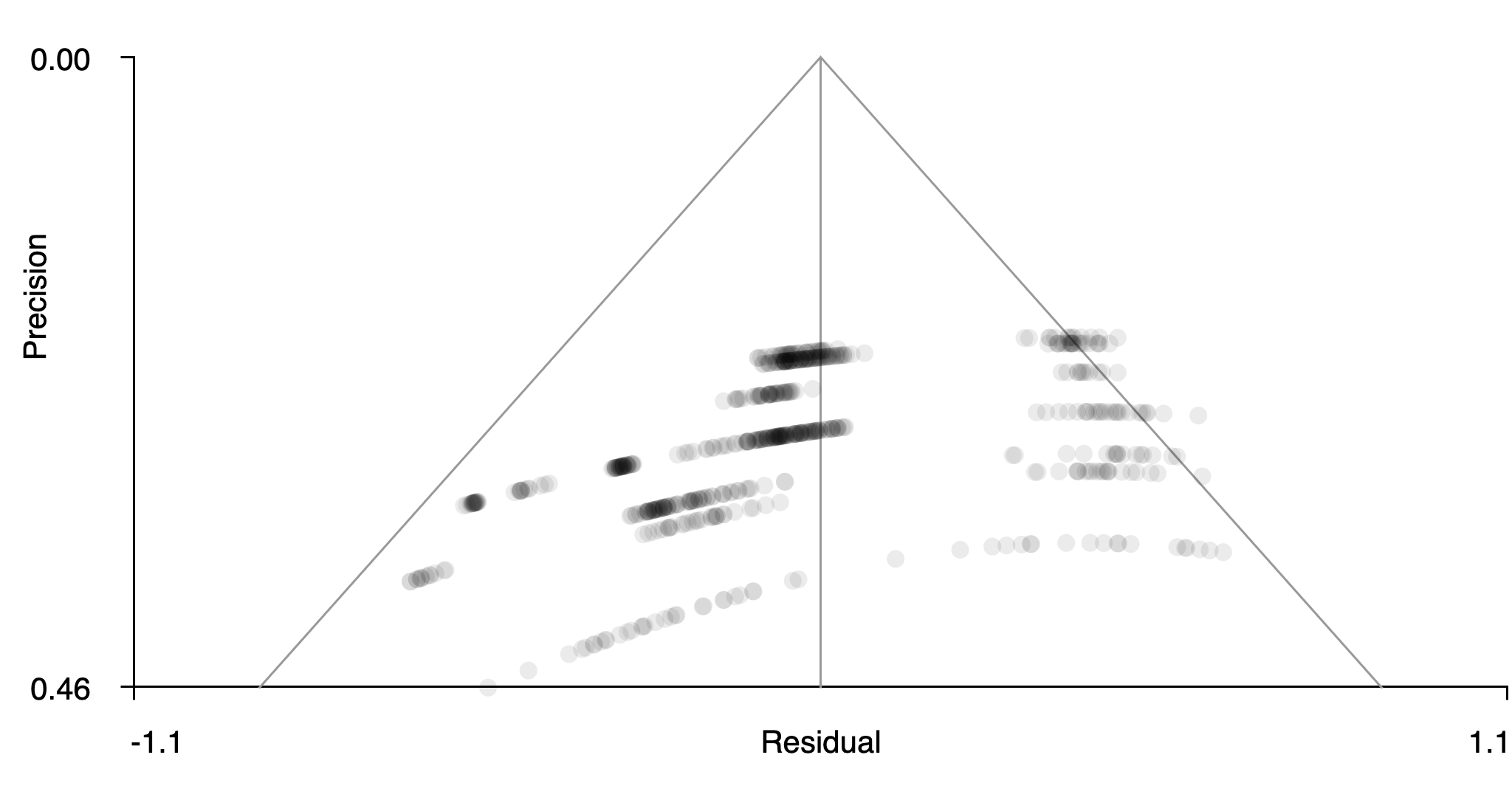
**

F

**Supplementary Tables**

**Supplementary Table 1:** Details of the tasks and selected contrasts for each included study.

| **Study** | **Task** | **Selected contrast** | **Stimuli** | **Category** |
| --- | --- | --- | --- | --- |
| Takahashi et al., 2002 | 1) Chinese characters were either pronounced but not presented visually or pronounced and presented visually; 2) During the scan, subjects judged whether the characters were perceived or imagined | imagined vs. perceived | verbal | Reality-monitoring |
| Turner et al., 2008 | 1) Subjects were presented with either a clue and target word or a clue and a target question mark prompting them to imagine the target word; 2) During the scan, subjects indicated whether words had been perceived or imagined | imagined vs. perceived | verbal | Reality-monitoring |
| King et al., 2014 | 1) Subjects were presented with either a word followed with a corresponding picture or a word followed with a black rectangle; 2) During the scan, subjects indicated whether the words corresponded to pictures that were perceived, imagined or new | imagined vs. perceived | verbal + picture | Reality-monitoring |
| Subramaniam et al., 2012 | 1) Subjects were shown sentences for which the final word was presented by the experimenter or left blank; 2) During the scan, subjects indicated whether words were externally presented or self-generated | self-generated vs. perceived | verbal | Reality-monitoring |
| Lundstrom et al., 2003 | 1) Subjects viewed either a word with a corresponding picture or a word followed by a blank screen; 2) During the scan, subjects indicated whether the words corresponded to pictures that were perceived or imagined | imagined vs. perceived | verbal | Reality-monitoring |
| King et al., 2017 | 1) Subjects viewed either a word with a corresponding picture or a word followed by a blank screen; 2) During the scan, subjects indicated whether the words corresponded to pictures that were perceived or imagined | imagined vs. perceived | verbal + picture | Reality-monitoring |
| King et al., 2015 | 1) Subjects viewed either a word with a corresponding picture or a word followed by a blank screen; 2) During the scan, subjects indicated whether the words corresponded to pictures that were perceived or imagined | imagined vs. perceived | verbal + picture | Reality-monitoring |
| Vinogradov et al., 2008 | 1) Subjects were shown sentences for which the final word was presented by the experimenter or left blank; 2) During the scan, subjects indicated whether words were externally presented or self-generated | self-generated vs. perceived | verbal | Reality-monitoring |
| Stephan-Otto et al., 2017 | 1) Subjects viewed either a word with a corresponding picture or a word followed by a blank screen; 2) During the scan, subjects indicated whether the words corresponded to pictures that were perceived or imagined | imagined vs. perceived | verbal + picture | Reality-monitoring |
| Tsakiris et al., 2010 | Subjects viewed a video image of their right hand that was covered with a woolen glove. This image was either direct or delayed. In the passive condition, an experimenter lifted and lowered the index finger up and down. In the active condition, the subject actively lifted and lowered his/her finger. | synchronous vs. asynchronous | action - visual feedback | Self-monitoring |
| Uhlmann et al., 2020 | Subjects held the handle of a device to perform movements. Subjects were presented with visual feedback on a screen. Subject's own hand or someone else's hand was displayed on the screen. Movements could either be self-generated of generated by the device. Videos were either presented in real time or delayed. Subsequently, subjects were required to indicate whether they detected a delay or not. | self-generated vs. externally generated | action - visual feedback | Self-monitoring |
| Renes et al., 2015 | Subjects performed a computerized task in which the color of a square was changed. They were required to indicate whether this change of color was computer-generated or self-generated. | self-generated vs. externally generated | action - visual feedback | Self-monitoring |
| Farrer et al., 2002 | Subjects traced a circle along a T-shaped path with a joystick. They were told that the circle would be drawn by either themselves or the experimenter. | self-generated vs. externally generated | action - visual feedback | Self-monitoring |
| Kontaris et al., 2009 | Subjects performed hand actions when receiving a visual feedback. The feedback was either compatible or incompatible from the actions they were executing. During the incompatible condition, subjects viewed a record of the movements generated by their hand in the preceding block when they performed a different sequence of movements. | synchronous vs. asynchronous | action - visual feedback | Self-monitoring |
| Sasaki et al., 2018 | Subjects performed finger movements while receiving visual feedback. The feedback varied in 3 factors: action kinematics, body identity and feedback timing. Subjects were instructed to judge the degree of congruity. | self-generated vs. externally generated | action - visual feedback | Self-monitoring |
| Schnell et al., 2007 | Subjects played a racing video game in which a car had to be kept on a racing track. Incongruity was artificially generated by intermittent takeover of the controls by the computer acting as an autopilot. Subjects were instructed to abstain from their own actions as soon as the computer took over control. | self-generated vs. externally generated | action - visual feedback | Self-monitoring |
| Jardri et al., 2007 | Subjects either listened to their own voice when whispering or listened to another person's voice. | self-generated vs. externally generated | verbal - verbal feedback | Self-monitoring |
| Jardri et al., 2011 | Subjects either listened their own voice when whispering or listened to another person's voice. | self-generated vs. externally generated | verbal - verbal feedback | Self-monitoring |
| Balslev et al., 2006 | Subjects held a joystick to execute some random movements. They were presented with visual feedback about their own movements, which was either vertical or distorted to a variable degree. During the passive condition, an experimenter moved the subject's fingers. During each session, subjects were asked to indicate if the movements they saw were synchronous, asynchronous, or actively or passively provoked. | synchronous vs. asynchronous | action - visual feedback | Self-monitoring |
| Farrer et al., 2008 | Subjects continuously performed finger movements. They were led to believe that they were watching alternating depictions of their own movements with a delay and those of another agent. They were asked to indicate whether they thought the observed movement was their own or belonged to another agent. | self-generated vs. externally generated | action - visual feedback | Self-monitoring |
| Farrer et al., 2003 | Subjects executed movements with a joystick. They were presented with either the actual feedback of their movement, the distorted feedback or the movement of the joystick controlled by another agent. Subjects were instructed to judge in the movement was their own movement, their own movement distorted or the movement of another agent. | Conjunction: synchronous vs. asynchronous and self-generated vs. externally generated | action - visual feedback | Self-monitoring |

**Supplementary Table 2:** Activation/deactivation peaks for the included studies in both meta-analyses (n = 21).

| **Authors** | **Coordinate system** | **ACTIVATIONS** | | | | |  |  | **DEACTIVATIONS** | | | | |  |  |
| --- | --- | --- | --- | --- | --- | --- | --- | --- | --- | --- | --- | --- | --- | --- | --- |
|  |  | **x** | **y** | **z** | **Z (peak)** | **t (peak)** | **F (peak)** | **p-value** | **x** | **y** | **z** | **Z (peak)** | **t (peak)** | **F**  **(peak)** | **p-value** |
| Takahashi et al., 2002 | TAL | 42 | -60 | 56 | 4.37 |  |  |  | -38 | -44 | -16 | 5.15 |  |  |  |
| Turner et al., 2008 | MNI | -39 | 45 | 24 | 3.48 |  |  |  |  |  |  |  |  |  |  |
|  |  | 12 | 48 | 27 | 3.76 |  |  |  |  |  |  |  |  |  |  |
|  |  | -33 | 27 | 42 | 4.28 |  |  |  |  |  |  |  |  |  |  |
|  |  | 45 | 15 | 12 | 4.31 |  |  |  |  |  |  |  |  |  |  |
|  |  | 57 | 12 | 42 | 3.76 |  |  |  |  |  |  |  |  |  |  |
|  |  | 33 | 39 | 45 | 4.53 |  |  |  |  |  |  |  |  |  |  |
|  |  | -30 | -6 | 57 | 3.97 |  |  |  |  |  |  |  |  |  |  |
|  |  | -9 | -18 | -51 | 3.62 |  |  |  |  |  |  |  |  |  |  |
|  |  | 36 | -9 | 36 | 3.82 |  |  |  |  |  |  |  |  |  |  |
|  |  | -51 | -36 | 9 | 4.12 |  |  |  |  |  |  |  |  |  |  |
|  |  | -63 | -45 | -6 | 3.66 |  |  |  |  |  |  |  |  |  |  |
|  |  | 57 | -57 | -12 | 3.63 |  |  |  |  |  |  |  |  |  |  |
|  |  | 60 | -27 | 15 | 4.01 |  |  |  |  |  |  |  |  |  |  |
|  |  | -39 | -36 | 54 | 4.14 |  |  |  |  |  |  |  |  |  |  |
|  |  | -63 | -33 | 24 | 3.63 |  |  |  |  |  |  |  |  |  |  |
|  |  | -42 | -63 | 42 | 4.5 |  |  |  |  |  |  |  |  |  |  |
|  |  | -3 | -54 | 27 | 4.1 |  |  |  |  |  |  |  |  |  |  |
|  |  | 42 | -81 | 33 | 4.24 |  |  |  |  |  |  |  |  |  |  |
|  |  | 6 | -84 | 42 | 3.62 |  |  |  |  |  |  |  |  |  |  |
|  |  | 18 | -69 | -30 | 4.47 |  |  |  |  |  |  |  |  |  |  |
|  |  | -15 | -57 | -27 | 3.56 |  |  |  |  |  |  |  |  |  |  |
| King et al., 2014 | MNI | 45 | -21 | 57 |  | 6.98 |  |  | -36 | -57 | 45 |  | 5.44 |  |  |
|  |  | 30 | 42 | 27 |  | 3.56 |  |  | -36 | 6 | 36 |  | 4.92 |  |  |
|  |  | -9 | -45 | 15 |  | 3.84 |  |  | 51 | 39 | 18 |  | 3.75 |  |  |
|  |  | -57 | -63 | 6 |  | 4.19 |  |  | -9 | 18 | 51 |  | 3.72 |  |  |
|  |  | 45 | 3 | -18 |  | 4.02 |  |  | -9 | -75 | 33 |  | 5.03 |  |  |
|  |  | -63 | -42 | 24 |  | 3.9 |  |  | -30 | -39 | -15 |  | 4.41 |  |  |
|  |  | 54 | -66 | 3 |  | 3.86 |  |  | -39 | -21 | 21 |  | 4.26 |  |  |
|  |  | 15 | -27 | 48 |  | 3.74 |  |  | -6 | -24 | 27 |  | 4.08 |  |  |
|  |  | 30 | -12 | 0 |  | 4.17 |  |  | 9 | 9 | 0 |  | 4.97 |  |  |
|  |  | -15 | -54 | -21 |  | 4.92 |  |  | 12 | -54 | -15 |  | 5.4 |  |  |
|  |  | -30 | -45 | -30 |  | 4 |  |  |  |  |  |  |  |  |  |
| Subramaniam et al., 2012 | MNI | 10 | 52 | 2 |  | 3.79 |  |  |  |  |  |  |  |  |  |
| Lundstrom et al., 2003 | TAL | -48 | 26 | 24 | 3.96 |  |  |  |  |  |  |  |  |  |  |
|  |  | -18 | -66 | 40 | 3.93 |  |  |  |  |  |  |  |  |  |  |
| King et al., 2017 | MNI |  |  |  |  |  |  |  | -48 | -52 | 28 |  |  | 22.47 |  |
|  |  |  |  |  |  |  |  |  | 60 | -25 | 28 |  |  | 21.17 |  |
| King et al., 2015 | MNI |  |  |  |  |  |  |  | -39 | -58 | 52 |  | 5.21 |  |  |
|  |  |  |  |  |  |  |  |  | 33 | -67 | 46 |  | 4.81 |  |  |
|  |  |  |  |  |  |  |  |  | -9 | -70 | 43 |  | 4.72 |  |  |
|  |  |  |  |  |  |  |  |  | -30 | 26 | -2 |  | 4.42 |  |  |
|  |  |  |  |  |  |  |  |  | 33 | 29 | 4 |  | 4.13 |  |  |
|  |  |  |  |  |  |  |  |  | -3 | 14 | 61 |  | 3.96 |  |  |
|  |  |  |  |  |  |  |  |  | 51 | 29 | 25 |  | 3.92 |  |  |
|  |  |  |  |  |  |  |  |  | -3 | -28 | 31 |  | 4.15 |  |  |
|  |  |  |  |  |  |  |  |  | -9 | -19 | -2 |  | 4.50 |  |  |
|  |  |  |  |  |  |  |  |  | 12 | 8 | 13 |  | 4.41 |  |  |
|  |  |  |  |  |  |  |  |  | -12 | 2 | 10 |  | 4.02 |  |  |
| Vinogradov et al., 2008 |  | -10 | 48 | 18 | 4.5 |  |  |  |  |  |  |  |  |  |  |
|  |  | -4 | 57 | 19 | 4.08 |  |  |  |  |  |  |  |  |  |  |
|  |  | _-4 | 49 | 12 | 3.87 |  |  |  |  |  |  |  |  |  |  |
|  |  | 2 | 40 | 20 | 3.74 |  |  |  |  |  |  |  |  |  |  |
|  |  | 8 | 59 | 19 | 3.63 |  |  |  |  |  |  |  |  |  |  |
|  |  | 6 | 52 | 21 | 3.61 |  |  |  |  |  |  |  |  |  |  |
|  |  | -8 | -56 | 36 | 4.3 |  |  |  |  |  |  |  |  |  |  |
| Stephan-Otto et al., 2017 |  |  |  |  |  |  |  |  | -16 | 3 | 23 |  |  |  | 0.001 |
|  |  |  |  |  |  |  |  |  | 10 | 2 | 13 |  |  |  | 0.001 |
|  |  |  |  |  |  |  |  |  | -7 | -91 | -1 |  |  |  | 0.021 |
| Tsakiris et al., 2010 | MNI | -22 | -54 | -24 | 4.44 |  |  |  | 52 | -38 | 38 | 4.53 |  |  |  |
|  |  | 24 | -40 | 54 | 4.15 |  |  |  | 40 | -58 | 26 | 4.82 |  |  |  |
|  |  | 38 | -42 | 58 | 3.72 |  |  |  | -38 | 20 | 2 | 4.01 |  |  |  |
|  |  | 12 | -48 | -20 | 3.69 |  |  |  | -16 | -84 | -26 | 3.88 |  |  |  |
|  |  | -44 | -18 | 18 | 3.56 |  |  |  | -12 | -62 | -38 | 3.85 |  |  |  |
|  |  | 24 | -72 | 36 | 3.46 |  |  |  | 40 | 52 | 14 | 3.8 |  |  |  |
|  |  |  |  |  |  |  |  |  | 24 | 48 | -14 | 3.56 |  |  |  |
|  |  |  |  |  |  |  |  |  | 50 | -46 | -2 | 3.51 |  |  |  |
|  |  |  |  |  |  |  |  |  | 60 | 20 | 6 | 3.5 |  |  |  |
| Uhlmann et al., 2020 | MNI | 10 | -94 | 10 |  | 6.63 |  |  | 18 | -48 | 42 |  | 6.79 |  |  |
|  |  | -6 | -66 | 6 |  | 6.1 |  |  | -8 | 34 | 44 |  | 6.57 |  |  |
|  |  | -12 | -80 | 10 |  | 5.03 |  |  | 18 | -46 | 58 |  | 6.41 |  |  |
|  |  |  |  |  |  |  |  |  | 44 | -16 | -6 |  | 5.51 |  |  |
|  |  |  |  |  |  |  |  |  | 52 | 0 | -8 |  | 4.12 |  |  |
|  |  |  |  |  |  |  |  |  | 20 | -58 | -50 |  | 4.07 |  |  |
|  |  |  |  |  |  |  |  |  | 62 | -8 | 22 |  | 3.78 |  |  |
|  |  |  |  |  |  |  |  |  | 62 | -10 | 32 |  | 3.65 |  |  |
|  |  |  |  |  |  |  |  |  | 54 | -10 | 36 |  | 3.52 |  |  |
| Renes et al., 2015 | MNI | -52 | -68 | 32 |  | 5.08 |  |  |  |  |  |  |  |  |  |
|  |  | -20 | 52 | 40 |  | 5.13 |  |  |  |  |  |  |  |  |  |
|  |  | 20 | 36 | 52 |  | 5.67 |  |  |  |  |  |  |  |  |  |
|  |  | 8 | 64 | 4 |  | 4.88 |  |  |  |  |  |  |  |  |  |
| Farrer et al., 2002 | TAL | 40 | 8 | 2 | 4.55 |  |  |  | 44 | -58 | 32 | 4.86 |  |  |  |
|  |  | -36 | -2 | 2 | 4.21 |  |  |  | -48 | -52 | 40 | 4.04 |  |  |  |
|  |  |  |  |  |  |  |  |  | -6 | -58 | 50 | 4.62 |  |  |  |
|  |  |  |  |  |  |  |  |  | 2 | -50 | 44 | 3.76 |  |  |  |
|  |  |  |  |  |  |  |  |  | -38 | 28 | 48 | 3.65 |  |  |  |
| Kontaris et al., 2009 | TAL | 24 | -85 | 1 |  | 9.21 |  |  | 57 | -55 | 19 |  | 7.8 |  |  |
|  |  | 24 | -76 | 16 |  | 5.72 |  |  | 60 | -46 | -5 |  | 9.59 |  |  |
|  |  |  |  |  |  |  |  |  | 45 | 12 | 22 |  | 6.65 |  |  |
|  |  |  |  |  |  |  |  |  | 48 | 17 | 2 |  | 6.04 |  |  |
|  |  |  |  |  |  |  |  |  | 9 | -55 | 34 |  | 6.2 |  |  |
|  |  |  |  |  |  |  |  |  | 42 | 5 | 34 |  | 6.17 |  |  |
|  |  |  |  |  |  |  |  |  | 0 | 38 | 43 |  | 6.94 |  |  |
|  |  |  |  |  |  |  |  |  | -54 | -52 | 16 |  | 8.81 |  |  |
|  |  |  |  |  |  |  |  |  | -6 | 8 | 55 |  | 5.22 |  |  |
|  |  |  |  |  |  |  |  |  | -45 | 11 | 31 |  | 6.21 |  |  |
|  |  |  |  |  |  |  |  |  | -42 | -46 | -8 |  | 8.86 |  |  |
|  |  |  |  |  |  |  |  |  | -42 | 20 | 2 |  | 6.1 |  |  |
| Sasaki et al., 2018 | MNI | 62 | -14 | 36 | 4.66 |  |  |  | -6 | 32 | 34 | 3.8 |  |  |  |
|  |  | 26 | -68 | 38 | 5.95 |  |  |  |  |  |  |  |  |  |  |
| Schnell et al., 2007 | TAL |  |  |  |  |  |  |  | 42 | 20 | -14 | 5.19 |  |  |  |
|  |  |  |  |  |  |  |  |  | 45 | 46 | -10 | 4.27 |  |  |  |
|  |  |  |  |  |  |  |  |  | 53 | 20 | -11 | 3.97 |  |  |  |
|  |  |  |  |  |  |  |  |  | 42 | 5 | 38 | 4.64 |  |  |  |
|  |  |  |  |  |  |  |  |  | 50 | 19 | 32 | 3.87 |  |  |  |
|  |  |  |  |  |  |  |  |  | 50 | 21 | 21 | 3.57 |  |  |  |
|  |  |  |  |  |  |  |  |  | 56 | 21 | 10 | 4.41 |  |  |  |
|  |  |  |  |  |  |  |  |  | 48 | 24 | 7 | 3.74 |  |  |  |
|  |  |  |  |  |  |  |  |  | 56 | -39 | 41 | 5.48 |  |  |  |
|  |  |  |  |  |  |  |  |  | 56 | -51 | 36 | 4.48 |  |  |  |
|  |  |  |  |  |  |  |  |  | 62 | -48 | 25 | 4.12 |  |  |  |
| Jardri et al., 2007 | TAL | 58 | -15 | 28 |  | 8.6 |  |  | 4 | 48 | 25 |  | 6.6 |  |  |
|  |  | -58 | -15 | 28 |  | 8.6 |  |  | -4 | 48 | 25 |  | 6.6 |  |  |
|  |  | -57 | -6 | 23 |  | 13 |  |  | -32 | 36 | 8 |  | 7.8 |  |  |
|  |  | 4 | -8 | 55 |  | 12.3 |  |  | 1 | -55 | 19 |  | 7.6 |  |  |
|  |  | 2 | 46 | 42 |  | 8.3 |  |  | -1 | -55 | 19 |  | 7.6 |  |  |
|  |  | -2 | 46 | 42 |  | 8.3 |  |  | 3 | 33 | 10 |  | 7.6 |  |  |
|  |  | -32 | 23 | 1 |  | 9.2 |  |  | -3 | 33 | 10 |  | 7.6 |  |  |
|  |  | -57 | 8 | 11 |  | 7.6 |  |  | 23 | -13 | -16 |  | 7.3 |  |  |
|  |  | 11 | -55 | -29 |  | 12.3 |  |  | 0 | -54 | 25 |  | 8.2 |  |  |
|  |  | -11 | -55 | -29 |  | 12.3 |  |  | 1 | 28 | 33 |  | 7.6 |  |  |
|  |  | 17 | -4 | 23 |  | 8.5 |  |  | -1 | 28 | 33 |  | 7.6 |  |  |
|  |  | -17 | -4 | 23 |  | 8.5 |  |  | 45 | -61 | 20 |  | 5.7 |  |  |
|  |  | 10 | -18 | 9 |  | 7.1 |  |  | -45 | -61 | 20 |  | 5.7 |  |  |
|  |  | -10 | -18 | 9 |  | 7.1 |  |  | 43 | -33 | 44 |  | 7.6 |  |  |
| Jardri et al., 2011 | TAL | 58 | -16 | 26 |  | 8.5 |  |  | 3 | -54 | 20 |  | 8 |  |  |
|  |  | -58 | -16 | 26 |  | 8.5 |  |  | -3 | -54 | 20 |  | 8 |  |  |
|  |  | -57 | -8 | 22 |  | 11.8 |  |  | 2 | -54 | 26 |  | 8.3 |  |  |
|  |  | 3 | -5 | 56 |  | 10.9 |  |  | 3 | 32 | 12 |  | 7.6 |  |  |
|  |  | 3 | 46 | 39 |  | 7.9 |  |  | -3 | 32 | 12 |  | 7.6 |  |  |
|  |  | -3 | 46 | 39 |  | 7.9 |  |  | 3 | 34 | 18 |  | 9.2 |  |  |
|  |  | -57 | 7 | 11 |  | 7.8 |  |  | -3 | 34 | 18 |  | 9.2 |  |  |
|  |  | 19 | -53 | -33 |  | 10.3 |  |  | 47 | -54 | 12 |  | 5.9 |  |  |
|  |  | -19 | -53 | -33 |  | 10.3 |  |  | -47 | -54 | 12 |  | 5.9 |  |  |
|  |  | 17 | -1 | 22 |  | 7.9 |  |  | 44 | -38 | 40 |  | 7.8 |  |  |
|  |  | -17 | -1 | 22 |  | 7.9 |  |  |  |  |  |  |  |  |  |
|  |  | 10 | -18 | 10 |  | 7.6 |  |  |  |  |  |  |  |  |  |
| Balslev et al., 2006 | MNI |  |  |  |  |  |  |  | 54 | -42 | 33 |  | 7.09 |  |  |
|  |  |  |  |  |  |  |  |  | -42 | -51 | 45 |  | 7.1 |  |  |
|  |  |  |  |  |  |  |  |  | -54 | -48 | 27 |  | 5.71 |  |  |
|  |  |  |  |  |  |  |  |  | 51 | -24 | -12 |  | 7.04 |  |  |
| Farrer et al., 2008 | MNI |  |  |  |  |  |  |  | 58 | -46 | 48 | 4.36 |  |  |  |
|  |  |  |  |  |  |  |  |  | 44 | -50 | 60 | 3.19 |  |  |  |
|  |  |  |  |  |  |  |  |  | -48 | -46 | 56 | 3.97 |  |  |  |
|  |  |  |  |  |  |  |  |  | -48 | 28 | 30 | 4.5 |  |  |  |
|  |  |  |  |  |  |  |  |  | -44 | 22 | 36 | 3.81 |  |  |  |
|  |  |  |  |  |  |  |  |  | 28 | 54 | -2 | 3.34 |  |  |  |
|  |  |  |  |  |  |  |  |  | 46 | 30 | 42 | 3.51 |  |  |  |
|  |  |  |  |  |  |  |  |  | -54 | 18 | 20 | 4.33 |  |  |  |
|  |  |  |  |  |  |  |  |  | 38 | 50 | -2 | 3.89 |  |  |  |
| Farrer et al., 2003 | MNI | 56 | -56 | 36 | 4.72 |  |  |  | 40 | -10 | 16 | 4.42 |  |  |  |
|  |  | -64 | -58 | 32 | 4.43 |  |  |  | 20 | -38 | -28 | 4.38 |  |  |  |
|  |  | 0 | 14 | 54 | 4.27 |  |  |  |  |  |  |  |  |  |  |
|  |  | 50 | 10 | 58 | 4.20 |  |  |  |  |  |  |  |  |  |  |
|  |  | 12 | 30 | 42 | 4.07 |  |  |  |  |  |  |  |  |  |  |

*Abbreviations*: MNI: Montreal Neurological 40Institute, TAL: Talairach

**Supplementary Table 3:** Imaging parameters and statistical threshold of each included study.

| **Authors** | **Magnetic field** | **Slice thickness, gap** | **Smoothing kernel** | **Number of slices** | **FOV** | **matrix** | **Threshold** | **design** |
| --- | --- | --- | --- | --- | --- | --- | --- | --- |
| Balslev et al., 2006 | 1.5T | 3 mm, NR | 8 mm | 42 | NR | 64x64 | p_uncorr_ < 0.001 | block |
| Farrer et al., 2002 | 2T | 3 mm, NR | 8 mm | 26 | 192 | 64x64 | p_uncorr_ < 0.0001 | event-related |
| Farrer et al., 2003 | Siemens CTI | NA | NA | 63 | 15.2 | NA | p_uncorr_ < 0.0001 | block |
| Farrer et al., 2008 | 1.5T | 4.5 mm, 1 mm | 8 mm | 25 | NR | NR | p_uncorr_ < 0.002 | block |
| Jardri et al., 2007 | 1.5T | 4 mm, NR | 4 mm | 30 | 240 | 64x64 | NR | block |
| Jardri et al., 2011 | 1.5T | 4 mm, NR | 5 mm | 30 | 240 | 64x64 | NR | block |
| King et al., 2014 | 3T | 3 mm, NR | 8 mm | 37 | NR | 64x64 | p_corr_ < 0.05 | event-related |
| King et al., 2015 | 3T | 3 mm, NR | 8 mm | 37 | NR | 64x64 | p_corr_ < 0.05 | event-related |
| King et al., 2017 | 3T | 3 mm, NR | 8 mm | 37 | NR | 64x64 | p_corr_ < 0.05 | event-related |
| Kontaris et al., 2009 | 3T | 3 mm, NR | no smoothing | 34 | NR | 64x64 | p_uncorr_ < 0.005 | block |
| Lundstrom et al., 2003 | 1.5T | 3 mm, 0.4 mm | 12 mm | 42 | NR | 64x64 | p_uncorr_ < 0.001 | event-related |
| Renes et al., 2015 | 3T | 4 mm, NR | 8 mm | 30 | 256x208 | 64x51 | p_uncorr_ < 0.001 | event-related |
| Sasaki et al., 2018 | 3T | 3 mm, NR | 8 mm | 39 | 192x192 | 64x64 | p_uncorr_ < 0.001 | block |
| Schnell et al., 2007 | 1.5T | 4 mm, 0.2 mm | 9 mm | 30 | 192 | 64x64 | p_corr_ < 0.01 | event-related |
| Stephan-Otto et al., 2017 | 1.5T | 4 mm, 1 mm | 8 mm | 26 | 240 | 64x64 | p_corr_ < 0.05 | event-related |
| Subramaniam et al., 2012 | 3T | 6 mm, NR | 10 mm | 14 | 220 | 64x64 | p_uncorr_ < 0.001 | event-related |
| Takahashi et al., 2002 | 1.5T | 6 mm, NR | 8 mm | 20 | 256 | 64x64 | p_corr_ < 0.05 | event-related |
| Tsakiris et al., 2010 | 1.5T | 3 mm, NR | 8 mm | 48 | NR | NR | p_uncorr_ < 0.001 | block |
| Turner et al., 2008 | 3T | 2 mm, 1 mm | 8 mm | 36 | NR | 64x64 | p_uncorr_ < 0.001 | event-related |
| Uhlmann et al., 2020 | 3T | 4 mm, 0.6 mm | 8 mm | 34 | 192 | 64x64 | p_uncorr_ < 0.001 | block |
| Vinogradov et al., 2008 | 1.5T | 5 mm, 1mm | 8 mm | 19 | 26x26 | 128x128 | p_uncorr_ < 0.001 | event-related |

*Abbreviations*: FOV: Field of view; NR: Not Reported; T: Tesla.

**Supplementary Table 4:** Quality assessment checklist. Score 0/0.5/1 for each item (0.5 points were given for criteria partially met).

| **Subjects** |
| --- |
| 1. The sample size was appropriate |
| 2. Subjects were evaluated prospectively, demographic data were reported (age (mean and SD/range), sex, and handedness), and psychiatric and medical illnesses were excluded |
| 3. If any subject was scanned but then rejected from the analysis, withdrawals from the study were explained |
| **Methods for self-recognition tasks** |
| 4. All participants went through a training session outside the scanner |
| 5. The design was clearly described so that it could be reproduced (number of blocks or trials per subject, length of each trial and ISI, block or event-related design) |
| 6. The stimuli and the number of repetitions were sufficient and clearly described |
| 7. If applicable, the baseline condition was defined as almost the same as the task condition except for the self-recognition |
| **Methods for image acquisition and statistical analysis** |
| 8. MRI slice thickness ≤ 3 mm |
| 9. 3T MRI was used |
| 10. The imaging technique used for data acquisition was clearly described so that it could be reproduced (e.g., MRI system used, field strength, pulse sequence type, number of volumes per session, field of view, matrix size, slice thickness, interslice skip, acquisition orientation, TE/TR/flip angle) |
| 11. Preprocessing operations were clearly described and detailed so that they could be reproduced (e.g., software used, order of preprocessing operations, slice-timing, motion correction, coregistration and normalization (linear/affine or nonlinear), smoothing) |
| 12. Adjustments were made for multiple statistical comparisons |
| 13. Appropriate design and/or analytical methods to control confounding |
| 14. Appropriate use of statistics for primary analysis effect (excluding control of confounders) |
| **Results, conclusions, and conflicts of interest** |
| 15. Statistical parameters for significant and important nonsignificant differences were provided |
| 16. Conclusions were consistent with the results obtained and the limitations were discussed |
| 17. Declarations of conflicts of interest or identification of funding sources |

**Supplementary Table 5:** Sensitivity analysis for the reality-monitoring meta-analysis

|  | Activation | | | Deactivation | | | | | |
| --- | --- | --- | --- | --- | --- | --- | --- | --- | --- |
| Study | Left cerebellum, hemispheric lobule VI | Right superior frontal gyrus, medial (BA10) | Left supramarginal gyrus (BA 48) | Right anterior thalamic projections | Left medial cingulate (BA 23) | Right inferior frontal gyrus, triangular part | Left precuneus (BA 7) | Left caudate nucleus | Left supplementary motor area (BA 6) |
| King et al., 2014 | n | y | n | y | n | n | n | y | n |
| King et al., 2015 | y | y | y | y | n | n | n | n | n |
| King et al., 2017 | y | y | y | y | y | y | y | y | y |
| Stephan-Otto et al., 2017 | y | y | y | y | y | y | y | y | y |
| Subramaniam et al., 2012 | y | y | y | y | y | y | y | y | y |
| Takahashi et al., 2002 | y | y | y | y | y | y | n | y | y |
| Turner et al., 2008 | n | y | n | y | y | y | y | y | y |
| Lundstrom et al., 2003 | y | y | n | y | y | y | y | y | y |
| Vinogradov et al., 2008 | y | n | y | y | y | y | y | y | y |
| **Total** | **7/9** | **8/9** | **6/9** | **9/9** | **7/9** | **7/9** | **6/9** | **8/9** | **7/9** |

**Supplementary Table 6:** Sensitivity analysis for the self-monitoring meta-analysis

|  | Activation | | | | Deactivation | | | | | | | | |
| --- | --- | --- | --- | --- | --- | --- | --- | --- | --- | --- | --- | --- | --- |
| Study | Left cerebellum, hemispheric lobule VI | Left postcentral gyrus (BA 48) | Corpus callosum | Right supplementary motor area (BA 6) | Right supramarginal gyrus (BA 22) | Right precuneus | Left superior frontal gyrus (BA 32) | Left inferior parietal gyri (BA 40) | Right inferior frontal gyrus, opercular part (BA 48) | Left inferior frontal gyrus, triangular part (BA 48) | Right anterior cingulate / paracingulate gyri (BA 11) | Right middle temporal gyrus (BA 48) | Right middle temporal gyrus (BA 21) |
| Balslev et al., 2006 | y | y | y | y | y | y | y | y | y | y | n | n | y |
| Farrer et al., 2002 | y | y | y | y | y | y | y | y | n | y | y | y | y |
| Farrer et al., 2003 | y | y | y | y | y | y | y | y | y | y | y | y | y |
| Farrer et al., 2008 | y | y | y | y | y | y | y | y | y | n | y | y | y |
| Jardri et al., 2007 | y | y | y | n | y | y | y | y | y | y | n | y | n |
| Jardri et al., 2011 | y | y | y | n | y | y | y | y | y | y | n | y | y |
| Kontaris et al., 2009 | y | y | y | y | y | y | y | y | n | y | n | y | n |
| Renes et al., 2015 | y | y | y | y | y | y | y | y | n | y | n | y | y |
| Sasaki et al., 2018 | y | y | y | y | y | y | y | y | y | y | y | n | y |
| Schnell et al., 2007 | y | y | y | y | y | y | y | y | n | n | y | n | y |
| Tsakiris et al., 2010 | y | y | y | y | y | y | y | y | n | y | y | n | y |
| Uhlmann et al., 2020 | y | y | y | y | y | y | y | y | y | y | y | n | y |
| **Total** | **12/12** | **12/12** | **12/12** | **10/12** | **12/12** | **12/12** | **12/12** | **12/12** | **7/12** | **10/12** | **7/12** | **7/12** | **10/12** |

**Supplementary Table 7:** Results of meta-regression for the reality-monitoring meta-analysis.

| **Variable** | **Brain region** | **BA** | **Number of voxels** | **p-value** | **MNI** | **SDM-Z** |
| --- | --- | --- | --- | --- | --- | --- |
| **Age***, increase* | ns |  |  |  |  |  |
|  | ns |  |  |  |  |  |
| **Age***, decrease* | ns |  |  |  |  |  |
| **Quality score***, increase* | ns |  |  |  |  |  |
| **Quality score,** *decrease* | ns |  |  |  |  |  |

The results are displayed based on the familywise error rate correction threshold of p < 0.005. Coordinates are reported in Montreal Neurological Institute (MNI) space.

*Abbreviations*: SDM-Z: Seed-based d Mapping Z-value; BA: Broadman Area.

**Supplementary Table 8:** Results of meta-regression for the self-monitoring meta-analysis.

| **Variable** | **Brain region** | **BA** | **Number of voxels** | **p-value** | **MNI** | **SDM-Z** |
| --- | --- | --- | --- | --- | --- | --- |
| **Age***, increase* | Left inferior frontal gyrus, opercular part | 44 | 116 | 0.00199 | -54, 14, 12 | 3.142 |
|  | Right supplementary motor area |  | 11 | 0.00400 | 4, 12, 54 | 3.117 |
| **Age***, decrease* | ns |  |  |  |  |  |
| **Quality score***, increase* | ns |  |  |  |  |  |
| **Quality score,** *decrease* | ns |  |  |  |  |  |

The results are displayed based on the familywise error rate correction threshold of p < 0.005. Coordinates are reported in Montreal Neurological Institute (MNI) space.

*Abbreviations*: SDM-Z: Seed-based d Mapping Z-value; BA: Broadman Area.
